# Determinants of Total and Active Microbial Communities Associated with Cyanobacterial Aggregates in a Eutrophic Lake

**DOI:** 10.1128/msystems.00992-22

**Published:** 2023-03-16

**Authors:** Dezhuang Gao, Zhijie Chen, Junyi Zhang, Chao Wang, Yuan Ma, Jiahui Wang, Jie Deng

**Affiliations:** a Shanghai Key Lab for Urban Ecological Processes and Eco-Restorations, School of Ecological and Environmental Sciences, East China Normal University, Shanghai, China; b Jiangsu Wuxi Environmental Monitoring Center, Jiangsu, China; c Shanghai Institute of Eco-Chongming, Shanghai, China; University of East Anglia

**Keywords:** cyanobacterial aggregates, microbial activities, *Microcystis*, 16S rRNA, community assembly

## Abstract

Cyanobacterial aggregates (CAs) comprised of photosynthetic and phycospheric microorganisms are often the cause of cyanobacterial blooms in eutrophic freshwater lakes. Although phylogenetic diversity in CAs has been extensively studied, much less was understood about the activity status of microorganisms inside CAs and determinants of their activities. In this study, the 16S rRNA gene (rDNA)-based total communities within CAs in Lake Taihu of China were analyzed over a period of 6 months during the bloom season; the 16S rRNA-based active communities during daytime, nighttime, and under anoxic conditions were also profiled. Synchronous turnover of both cyanobacterial and phycospheric communities was observed, suggesting the presence of close interactions. The rRNA/rDNA ratio-based relative activities of individual taxa were predominantly determined by their rDNA-based relative abundances. In particular, high-abundance taxa demonstrated comparatively lower activities, whereas low-abundance taxa were generally more active. In comparison, hydrophysicochemical factors as well as diurnal and redox conditions showed much less impact on relative activities of microbial taxa within CAs. Nonetheless, total and active communities exhibited differences in community assembly processes, the former of which were almost exclusively controlled by homogeneous selection during daytime and under anoxia. Taken together, the results from this study provide novel insights into the relationships among microbial activities, community structure, and environmental conditions and highlight the importance of further exploring the regulatory mechanisms of microbial activities at the community level.

**IMPORTANCE** Cyanobacterial aggregates are important mediators of biogeochemical cycles in eutrophic lakes during cyanobacterial blooms, yet regulators of microbial activities within them are not well understood. This study revealed rDNA-based abundances strongly affected the relative activities of microbial taxa within *Microcystis* aggregates, as well as trade-off effects between microbial abundances and activities. Environmental conditions further improved the levels of relative activities and affected community assembly mechanisms in phycospheric communities. The relationships among microbial activities, abundances, and environmental conditions improve our understanding of the regulatory mechanisms of microbial activities in cyanobacterial aggregates and also provide a novel clue for studying determinants of microbial activities in other ecosystems.

## INTRODUCTION

Freshwater cyanobacterial blooms are worldwide threats to drinking water safety and lead to increased water treatment costs (1, 2). The bloom-forming cyanobacteria, including *Microcystis* and *Dolichospermum*, usually exist in the form of cyanobacterial aggregates (CAs) in natural water bodies (3). CAs are composed of both cyanobacterial cells and heterotrophic bacteria, aggregation of which is largely associated with algal production of exopolysaccharides (4, 5). Complicated interspecific relationships have been developed between cyanobacteria and phycospheric communities, forming hot spots of intricate interacting networks within the water column (6, 7). These interactions include both antagonism and mutualism, with the former exemplified by the isolation of numerous algicidal bacterial strains (8, 9) and the latter evidenced by discovery of a range of algal growth-promoting mechanisms: e.g., bacterially derived essential micronutrients such as vitamins, amino acids, and bioavailable trace metals (10–14). At the community level, the associations between cyanobacteria and phycospheric bacteria have also been investigated through profiling of community composition and analyses of ecological networks. The observed seasonal succession of CA-associated communities and the dynamics of heteroautotrophic network patterns have attracted much attention and suggested complex mechanisms of interspecific interactions within CAs (15, 16).

In addition to the long-term shift in the compositions and functions of CA-associated communities, intense metabolic changes in the short term also occur on a daily basis, largely due to circadian regulation of photoautotrophs and the associated changes in microscale chemical environments in the phycosphere. In CAs, daytime activities take place in the context of photosynthesis, during which time destructive reactive oxygen species are excessively produced (17), whereas removal of reactive oxygen species by phycospheric communities was found to be an important mechanism of heteroautotrophic interaction (18). Nighttime provides a relief of stress from UV exposure, yet meanwhile accompanies rewired carbon cycling among CA-associated microorganisms (19–22). The diurnal rhythm of microbial activities also induces sharp changes in redox conditions within CAs. During daytime, oxygenic photosynthesis generates hyperoxic centers inside individual aggregates, creating microniches with extremely high levels of oxidative stress (23), whereas during nighttime, respiration activities could expand hypoxic regions within aggregates, allowing the presence of inner anoxic spots that promote anaerobic metabolism (24–26). Indeed, transcriptomic studies of CAs have confirmed expression of genes associated with anaerobic metabolic pathways *in situ*, including the anaerobic nitrite reductase gene *nrfA*, N_2_O reductase gene *nosZ*, and the dissimilatory sulfite reductase gene *dsrA* (27–30). These pieces of evidence together suggest that highly fluctuating and diurnally regulated redox niches within CAs could provide important short-term regulators of activities and metabolic outcomes of CA-associated communities.

It is commonly acknowledged that long-term effects of environmental conditions could shape microbial community composition and structure, while short-term environmental changes or perturbation are more likely to affect temporary microbial activities and metabolism (31–33). In some limited studies, microbial activities may also vary with regard to their abundances. For example, Hunt et al. found that for marine microbial communities, activities were positively correlated with abundances in most cases (34), whereas Barreto et al. found that in an acidic aquatic environment, certain species could show opposite patterns in changes of activities and abundances (35); furthermore, a growing number of pieces of evidence suggested some rare microbial species may be surprisingly active and play important roles in biogeochemical cycling processes (36, 37). In freshwater ecosystems, CA-associated microbial communities were subjected to both long-term seasonal changes in hydrological and chemical conditions as well as short-term diurnal changes in biological activities and associated redox conditions. Although a range of studies have investigated compositional changes of CA-associated communities over time (15, 16, 38, 39), how long-term and short-term environmental conditions jointly affect activities of CA-associated communities and how microbial activities associate with abundance distribution patterns were rarely explored.

In this study, CAs were sampled at a single site of Lake Taihu, China, over a 6-month period during the bloom season. Both 16S rRNA gene (rDNA)-based profiling and 16S rRNA-based profiling were employed to study the dynamics of total and active CA-associated microbial communities (36, 37, 40, 41). Specifically, active CA-associated communities were characterized during daytime and nighttime, as well as under a dark anoxic condition, representing three types of short-term environmental fluctuation. This study aimed to answer the following questions. (i) What abiotic or biotic factors contributed to seasonal dynamics of cyanobacterial and the associated phycospheric microbial communities? (ii) How did activities of CA-associated communities relate to abundance distribution patterns? (iii) How did activities of CA-associated communities respond to long-term and short-term environmental changes? Answers to these questions shall provide insights into the drivers of community succession and determinants of microbial activities, which together, affect the dynamics of metabolic potential in CA-associated microbial communities.

## RESULTS

### Temporal changes of environmental factors.

During the course of sampling, the highest water temperature (WT) appeared in the end of July and the lowest WT appeared in the end of November. Electrical conductivity (EC) showed a decreasing trend from June to mid-September, yet with a sharp increase from then to the end of November. Water pH was mostly between 7 and 9 and was slightly higher during daytime than nighttime. Dissolved oxygen (DO) also exhibited significant diurnal variation, with daytime DO ranging from 6 mg/L to as high as over 12 mg/L and nighttime DO ranging from 4 mg/L to 11 mg/L. Dissolved inorganic nitrogen (DIN) fluctuated in the range of 0.5 mg/L to 3 mg/L, with nitrate-N (NO_3_-N) ranging from 0.02 mg/L to 1.2 mg/L. The concentrations of ammonia-N (NH4^+^-N) and nitrite-N (NO_2_-N) were comparatively lower. Abnormally high ammonia-N was observed on 27 June, when significant cyanobacterial decomposition and phycocyanin precipitation occurred at the end of a *Microcystis* bloom. Dissolved inorganic phosphate (DIP) was mostly in the range of 0.05 mg/L to 0.20 mg/L, with an abnormally high peak in the end of August, at which time DIN, nitrate-N, and nitrite-N also showed temporary peaks in values (see Fig. S1 in the supplemental material).

10.1128/msystems.00992-22.1 FIG S1 Hydrophysicochemical parameters during the sampling period. Download FIG S1, PDF file, 0.03 MB. Copyright © 2023 Gao et al. 2023 Gao et al. https://creativecommons.org/licenses/by/4.0/ This content is distributed under the terms of the Creative Commons Attribution 4.0 International license.

### Temporal dynamics of community composition in CAs.

**FIG 1:**
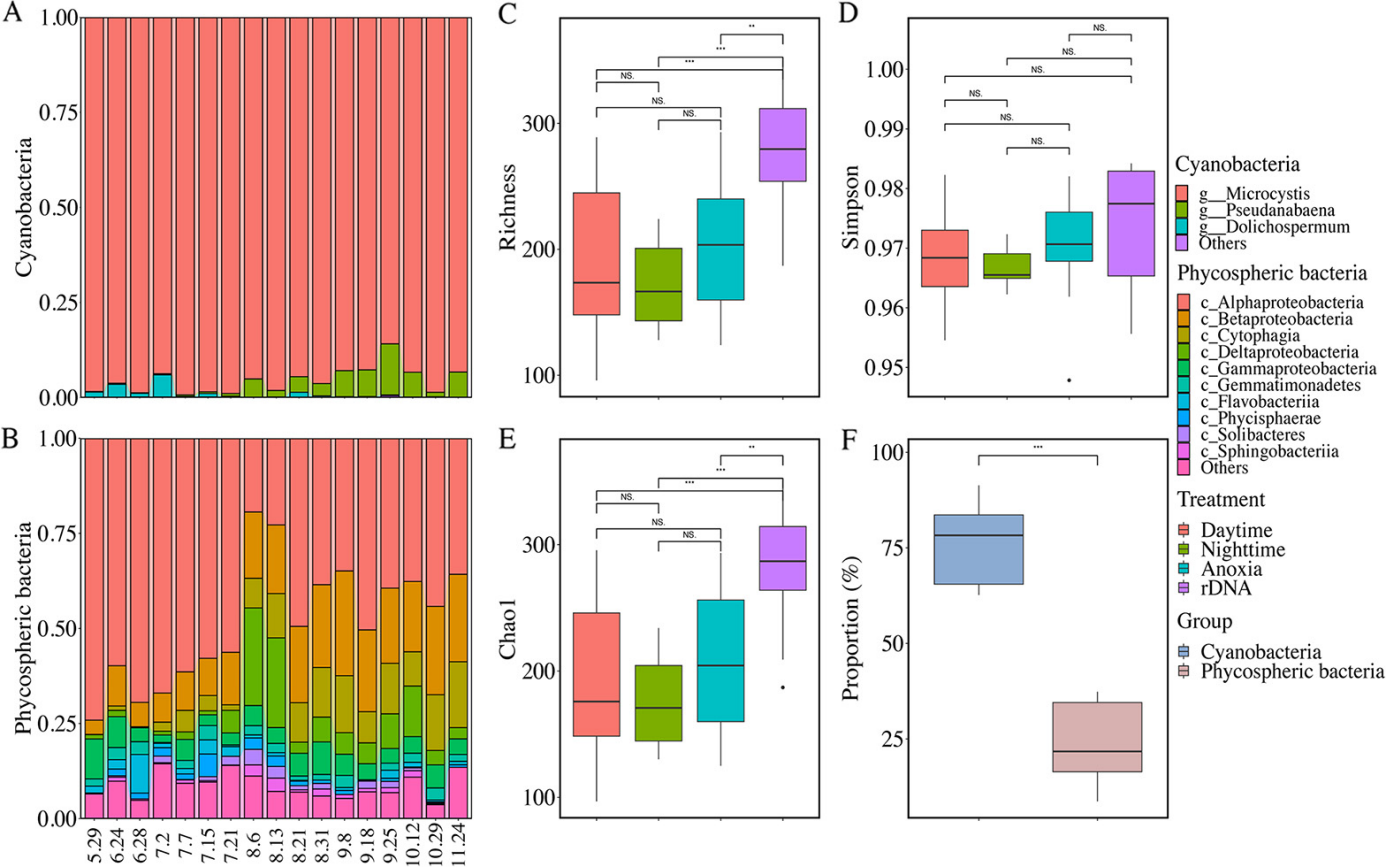
Community composition and α diversities in CAs. (A and B) Changes in composition of the dominant cyanobacterial (A) and phycospheric (B) communities during sampling; (C to F) α-diversity indices (richness, Simpson, and Chao1) of total and active communities under diurnal and anoxic conditions (C to E) and the rDNA-based abundance proportions of cyanobacterial and phycospheric communities (F).

Across all rDNA samples taken, cyanobacteria accounted for about 75% of the microbial communities in CAs (Fig. 1). *Microcystis* (*Chroococcales* order) was the most abundant genus of cyanobacteria, followed by *Dolichospermum* (*Nostocales* order) and *Pseudanabaena* (*Pseudanabaenales* order). Despite the dominance of *Microcystis* in all samples, *Dolichospermum* showed relatively greater abundances in June to July, and *Pseudanabaena* was relatively more abundant in the rest of the months. The most abundant phycospheric bacteria were those associated with *Alphaproteobacteria*, *Betaproteobacteria*, *Cytophagia*, Deltaproteobacteria, and *Gammaproteobacteria*, which together accounted for about 20% of total abundances. Specifically, the relative abundances of *Alphaproteobacteria* and *Flavobacteriia* generally decreased with time, whereas those of *Betaproteobacteria* and *Cytophagia* showed the opposite trend. Deltaproteobacteria showed markedly high abundances on 6 and 13 August. Classes including the *Gammaproteobacteria* and *Gemmatimonadetes* persisted and fluctuated throughout the sampling period.

**FIG 2:**
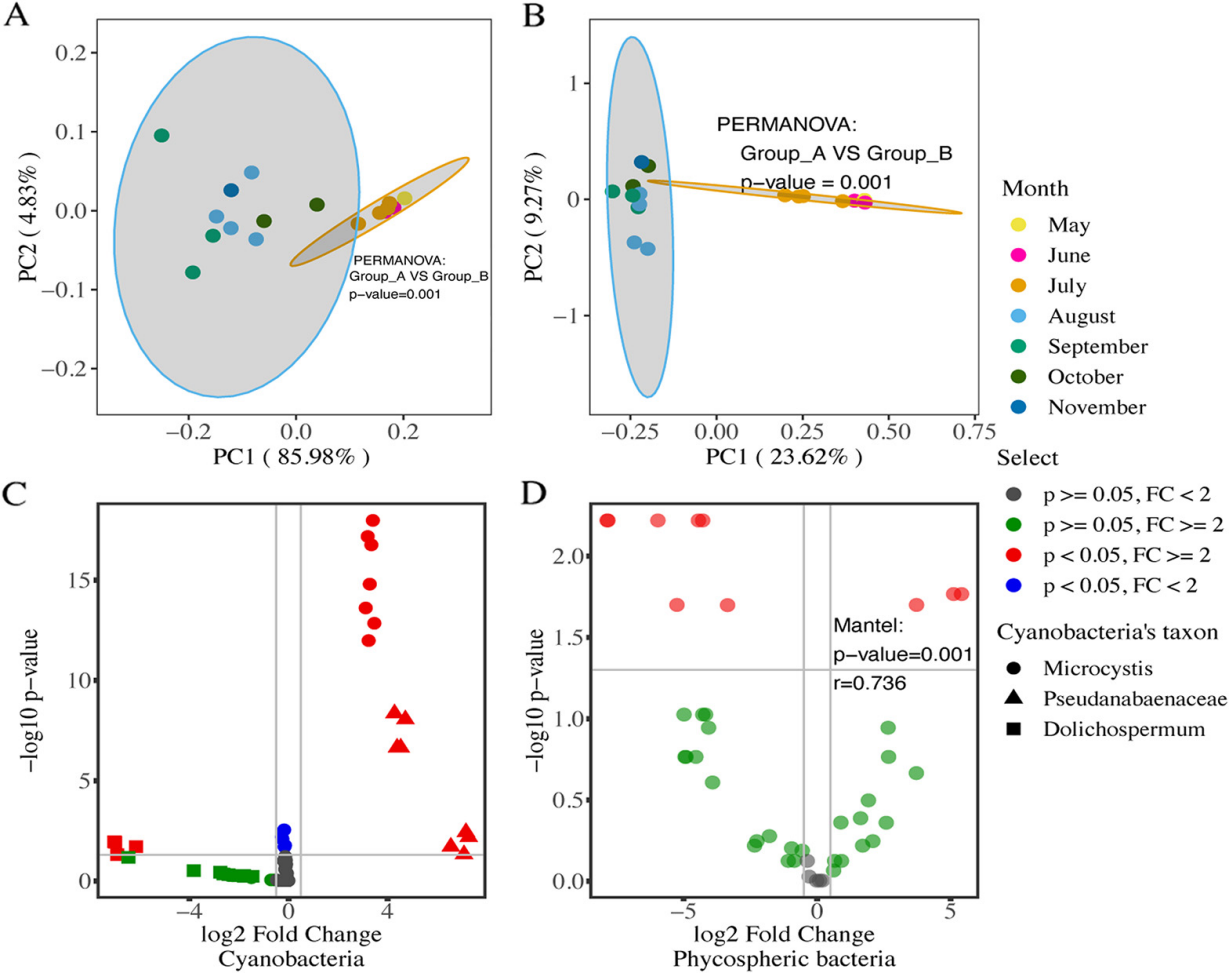
Temporal partitioning of cyanobacterial and phycospheric communities. (A and B) PCoA for phycospheric communities (A) and cyanobacterial (B) communities based on the Bray-Curtis distance matrices; (C and D) differentially distributed cyanobacterial ASVs (C) and phycospheric bacterial genera (D) between the two time periods (from May to July and from August to November) by DESeq2 analysis.

For both cyanobacterial and phycospheric communities, principal-coordinate analysis (PCoA) revealed apparent partitioning of samples with respect to sampling time along the first coordinate, which alone accounted for 86.0% and 23.6% of total variation (Fig. 2A and B). Samples taken in May to July clustered toward the right, whereas those taken in September to November distributed toward the left, and there were significant differences in both cyanobacterial and phycospheric communities between the two groups of samples (*P* < 0.01). DESeq analysis further revealed a range of amplicon sequence variants (ASVs) associated with *Microcystis* and phycospheric bacterial genera, whose abundances varied between the two sampling periods (Fig. 2C and D). The Mantel test revealed significant associations between the turnover of phycospheric communities with that of total cyanobacterial communities (*P* < 0.01; *r* = 0.540) and with that of *Microcystis* populations (*P* < 0.01; *r* = 0.517) (Table S1). Particularly, for the cyanobacterial and phycospheric bacterial taxa that showed significant temporal partitioning patterns, significant association in their turnover patterns was also revealed (Mantel test; *P* < 0.01). Hence, turnover of cyanobacteria, particularly *Microcystis* populations, provided an important driver of succession of phycospheric communities in CAs.

10.1128/msystems.00992-22.9 TABLE S1 Mantel analysis of phycospheric community composition with environmental factors and cyanobacterial community composition. Download Table S1, DOCX file, 0.02 MB. Copyright © 2023 Gao et al. 2023 Gao et al. https://creativecommons.org/licenses/by/4.0/ This content is distributed under the terms of the Creative Commons Attribution 4.0 International license.

To assess how cyanobacteria shaped the composition of phycospheric communities, we further looked for specific associations between major bacterial groups and cyanobacterial populations. In general, *Microcystis* and *Pseudanabaena* populations showed opposite trends of correlations with most bacterial taxa. For instance, relative abundances of *Burkholderiales*, *Cytophagales*, *Bdellovibrionales*, and *Caulobacterales* were negatively correlated with those of the dominant *Microcystis* ASVs yet positively correlated with those of *Pseudanabaena* ASVs, whereas taxa including *Sphingomonadales*, *Rhodospirillales*, and *Gemmatimonadales* showed the opposite pattern. Meanwhile, bacterial taxa including *Enterobacteriales*, *Actinomycetales*, and *Desulfarculales*, etc., showed positive associations with *Dolichospermum* in their relative abundances (Fig. S2).

10.1128/msystems.00992-22.2 FIG S2 Relationships between dominant cyanobacterial ASVs and phycospheric bacterial taxa. The Spearman’s correlation coefficients, ranging from −1 to 1, were displayed as colors ranging from blue to red. Cya_M, Cya_P, and Cya_D correspond to ASVs associated with *Microcystis*, *Pseudanabaenaceae*, and *Dolichospermum*, respectively. Ranges of *P* values are shown as follows: ***, *P* < 0.001; **, 0.001 ≤ *P* < 0.01; *, 0.01 ≤ *P* < 0.05. Download FIG S2, PDF file, 0.01 MB. Copyright © 2023 Gao et al. 2023 Gao et al. https://creativecommons.org/licenses/by/4.0/ This content is distributed under the terms of the Creative Commons Attribution 4.0 International license.

Environmental factors also significantly impacted the dynamics of cyanobacterial and phycospheric communities and partially contributed to their temporal partitioning patterns. Redundancy analysis (RDA) revealed that the abundances of the major ASVs of *Microcystis* and *Dolichospermum* were positively associated with DIN and EC, while the three main ASVs of *Pseudoanabaena* were positively correlated with nitrite-N and DIP. For phycospheric bacteria, *Sphingomonadales* were strongly associated with EC and ammonium-N, and *Spirobacillales* was better associated with turbidity (Tur) and nitrite-N, whereas *Burkholderiales* and *Cytophagales* were more influenced by DIP (Fig. S3).

10.1128/msystems.00992-22.3 FIG S3 Redundancy analysis (RDA) of cyanobacterial and phycospheric community composition with environmental factors. Colors represent sampling months, shapes represent three different conditions, and solid blue lines represent the relationships between dominant taxa and environmental factors. Download FIG S3, PDF file, 0.01 MB. Copyright © 2023 Gao et al. 2023 Gao et al. https://creativecommons.org/licenses/by/4.0/ This content is distributed under the terms of the Creative Commons Attribution 4.0 International license.

### Determinants of relative activities in CA-associated communities.

**FIG 3:**
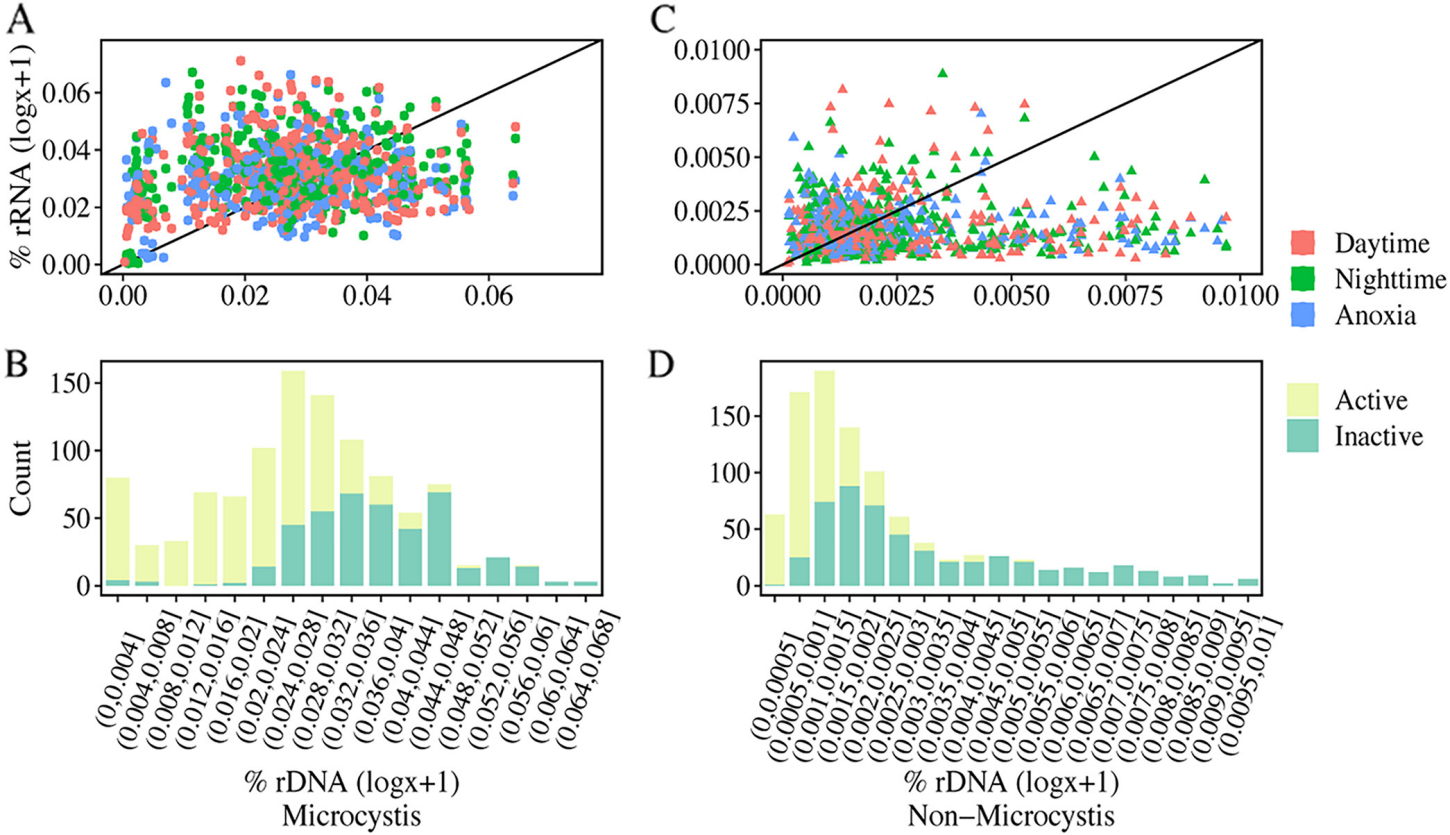
Relationship between 16S rRNA- and 16S rDNA-based abundances in CAs. (A to D) Relationship between 16S rRNA and 16S rDNA of *Microcystis* (A) and non-*Microcystis* communities (C) and the distributions of their activity states (B and D). The black solid line represents a 1:1 contour, and the colors of the dots represent diurnal and anoxic conditions. Note that this analysis only considered ASVs with nonzero values of rDNA-based and rRNA-based relative abundances.

**FIG 4:**
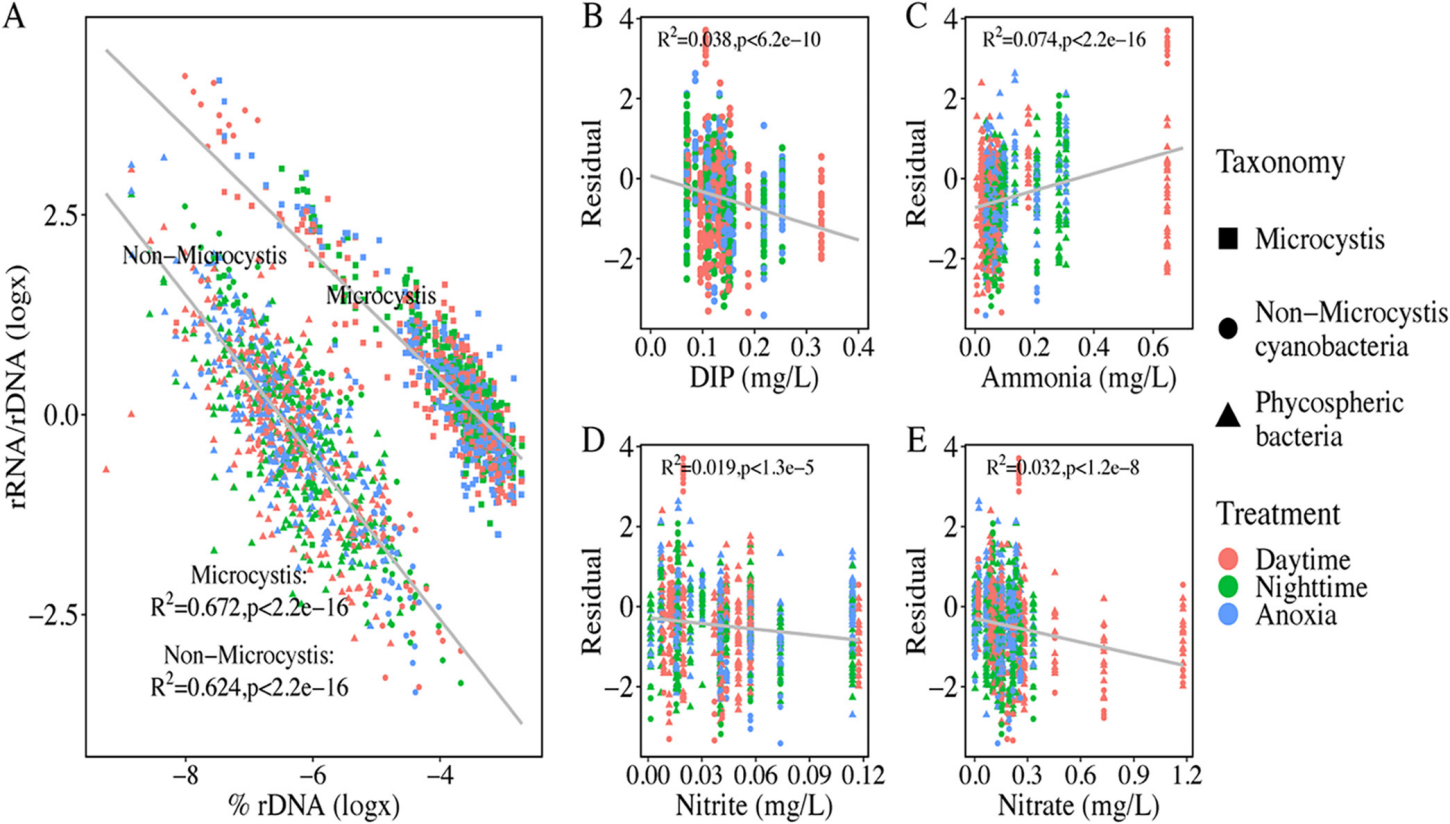
Relationships between 16S rRNA/rDNA-based relative activities and rDNA-based relative abundances as well as environmental factors. (A to E) Relationships between relative activities and rDNA-based relative abundances (A) and the effect of environmental factors on the residuals from the above regression analysis (B to E). The colors of the dots represent diurnal and anoxic conditions, and the shapes represent different groups of the CA-associated communities. Note that this analysis only considered ASVs with nonzero values of rDNA-based and rRNA-based relative abundances.

Active bacterial communities in cyanobacterial aggregates were characterized using rRNA-based abundances under diurnal and anoxic conditions. Both richness and Chao1 indices of the rRNA-based bacterial communities were significantly lower than those based on rDNA (Fig. 1). For both *Microcystis* and non-*Microcystis* communities, positive correlations between individual 16S rRNA- and rDNA-based relative abundances were revealed (Fig. 3). In the scatterplot of 16S rRNA- and rDNA-based relative abundances, dots that positioned above the 1:1 line indicated relatively active taxa, whereas those positioning below the 1:1 line indicated nonactive taxa. Interestingly, at low abundances, greater proportions of active taxa were found, while at high abundances, most taxa were nonactive. This pattern held for both *Microcystis* and non-*Microcystis* communities (Fig. 3). The rRNA/rDNA ratios were further used to evaluate the relative activities of microbial taxa, which showed a strong and negative correlation with rDNA-based abundances (Fig. 4A): i.e., low-abundance taxa were of greater relative activities, whereas high-abundance taxa were less active. This pattern was present under both diurnal and anoxic conditions (Fig. S4) and across dominant bacterial taxa (Fig. S5). Notably, *Microcystis*-associated ASVs were clearly distinguished from all other ASVs and exhibited higher relative activities at the same abundance levels, with the exception of a few *Nostocales*-associated ASVs showing extremely high relative activities (Fig. 4A). Remarkably, rDNA-based abundances explained 67% and 62% of total variance in the relative activities of *Microcystis* and non-*Microcystis* communities, respectively, and hence, were the strongest predictor of relative activities in CA-associated communities.

10.1128/msystems.00992-22.4 FIG S4 relationships between rRNA/rDNA-based relative activities and rDNA-based relative abundances (A) and effect of environmental factors on the residuals (B to E) under three conditions. Colors of the dots represent sampling months, and shapes of dots represent different groups of CA-associated communities. Note that this analysis only considered ASVs whose rDNA- and rRNA-based abundances were not zero. Download FIG S4, PDF file, 0.1 MB. Copyright © 2023 Gao et al. 2023 Gao et al. https://creativecommons.org/licenses/by/4.0/ This content is distributed under the terms of the Creative Commons Attribution 4.0 International license.

10.1128/msystems.00992-22.5 FIG S5 Relationships between rRNA/rDNA-based relative activities and rDNA-based relative abundances for the dominant microbial taxa within CAs. Colors of the dots represent sampling months. Note that this analysis only considered ASVs whose rDNA- and rRNA-based abundances were not zero. Download FIG S5, PDF file, 0.1 MB. Copyright © 2023 Gao et al. 2023 Gao et al. https://creativecommons.org/licenses/by/4.0/ This content is distributed under the terms of the Creative Commons Attribution 4.0 International license.

**FIG 5:**
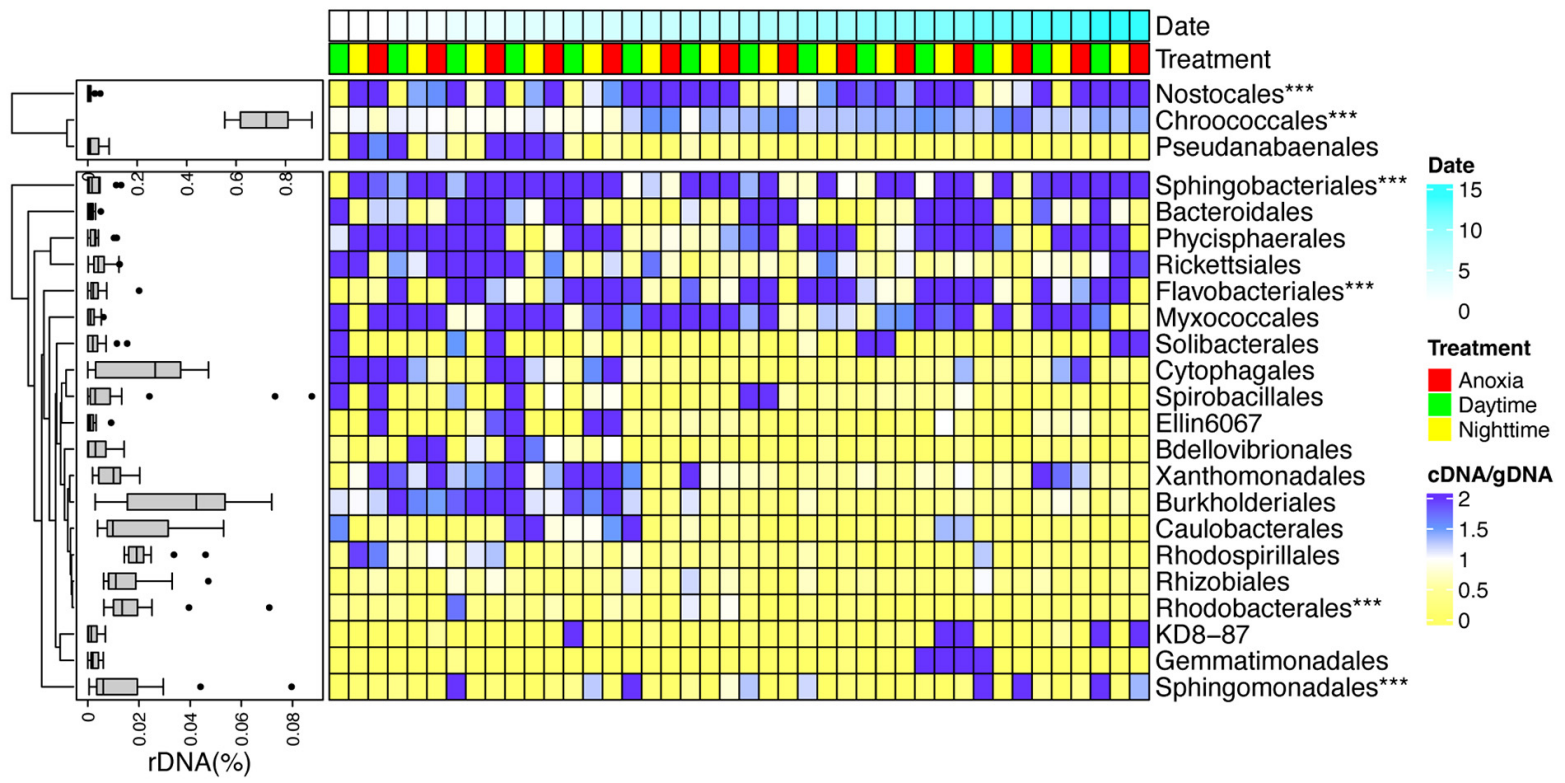
Dynamics of relative activities of dominant orders in CAs. The box plot on the left illustrates the rDNA-based relative abundances of each order, and *** on the right indicates significant differences among diurnal and anoxic conditions based on MRPP (multiple-response permutation procedure) analysis (with strata set by sampling dates).

Based on our finding that CA-associated communities exhibited significant temporal turnover patterns, we specifically examined their temporal dynamics of relative activities and the association with rDNA-based abundances. Indeed, for the dominant bacterial taxa, the temporal distribution of relative activities was driven, at least in part, by that of relative abundances (Fig. S5). For example, phycospheric bacterial lineages including *Cytophagales* and *Burkholderiales* showed higher relative activities during the early sampling stage (Fig. 5), whereas lineages including *Sphingomonadales* showed increased relative activities toward the end of sampling stage. The higher relative activities of the above taxa all occurred during the period when low rDNA-based abundances were observed (Fig. S6). Such a pattern was also supported in the dominant cyanobacteria *Chroococcales*, whose relative activity increased and rDNA-based abundance decreased toward the later sampling period, whereas *Pseudoanabaenaeles* showed the opposite trend (Fig. 1A). These results suggested the temporal distribution of relative activities of CA-associated taxa could be partly predicted by temporal distribution of their rDNA-based abundances.

10.1128/msystems.00992-22.6 FIG S6 Variation in rDNA-based relative abundances of dominant phycospheric taxa during the course of sampling. Download FIG S6, PDF file, 0.03 MB. Copyright © 2023 Gao et al. 2023 Gao et al. https://creativecommons.org/licenses/by/4.0/ This content is distributed under the terms of the Creative Commons Attribution 4.0 International license.

Environmental factors explained an additional, though minor, part of variation in relative activities of CA-associated communities. Here, the residuals of relative activities with rDNA-based abundances were further regressed against hydrophysicochemical parameters. Significant correlations were revealed for non-*Microcystis* communities with multiple parameters (Fig. 4B to E). Specifically, DO, nitrate-N, ammonia-N, and DIP were of the greatest impact according to random forest analysis, while the importance ranking slightly varied among diurnal and anoxic conditions (Fig. S7). Correlations between environmental factors and residuals for individual dominant bacterial taxa were also assessed (Fig. S8). However, no significant correlations with environmental factors could be found for residuals from *Microcystis*-associated ASVs. Collectively, the above results suggested relative activities of non-*Microcystis* communities were jointly shaped by both rDNA-based abundances and environmental factors, yet relative activities of *Microcystis* populations were solely affected by rDNA-based abundances.

10.1128/msystems.00992-22.7 FIG S7 The importance of environmental factors to the residuals from regression of rRNA/rDNA ratios with rDNA-based relative abundances for non-*Microcystis* communities under all conditions (A) and under each condition (B to D). The *P* values are represented as follows: ***, *P* < 0.001; **, 0.001 ≤ *P* < 0.01; *, 0.01 ≤ *P* < 0.05. Download FIG S7, PDF file, 0.01 MB. Copyright © 2023 Gao et al. 2023 Gao et al. https://creativecommons.org/licenses/by/4.0/ This content is distributed under the terms of the Creative Commons Attribution 4.0 International license.

10.1128/msystems.00992-22.8 FIG S8 Relationships between the residuals (from regression of rRNA/rDNA ratios with rDNA-based relative abundances) and environmental factors for dominant microbial groups. Colors of the dots represent different sampling months. Note that this analysis only considered ASVs whose rDNA- and rRNA-based abundances were not zero. Download FIG S8, PDF file, 0.2 MB. Copyright © 2023 Gao et al. 2023 Gao et al. https://creativecommons.org/licenses/by/4.0/ This content is distributed under the terms of the Creative Commons Attribution 4.0 International license.

### Relative activities of microbial communities in CAs under diurnal and anoxic conditions.

Bacterial groups whose relative activities varied among diurnal or anoxic conditions were further examined, and significant differences were revealed in only a few taxa (Fig. 5; Table S2). For the dominant cyanobacteria, *Chroococcales* showed a slight decrease in relative activities under anoxia; *Nostocales* showed comparatively lower activities during both nighttime and anoxia. Among phycospheric bacteria, *Sphingomonadales*, *Bacteroidales*, and *Rhodobacterales* exhibited relatively higher activities under anoxia in most time points.

10.1128/msystems.00992-22.10 TABLE S2 Orders with significantly different relative activities among the three conditions by MRPP analysis and paired Wilcoxon test. The *P* values are represented as follows: ***, *P* < 0.001; **, 0.001 ≤ *P* < 0.01; *, 0.01 ≤ *P* < 0.05. Download Table S2, DOCX file, 0.02 MB. Copyright © 2023 Gao et al. 2023 Gao et al. https://creativecommons.org/licenses/by/4.0/ This content is distributed under the terms of the Creative Commons Attribution 4.0 International license.

### Community assembly processes on active phycospheric communities under diurnal and anoxic conditions.

**FIG 6:**
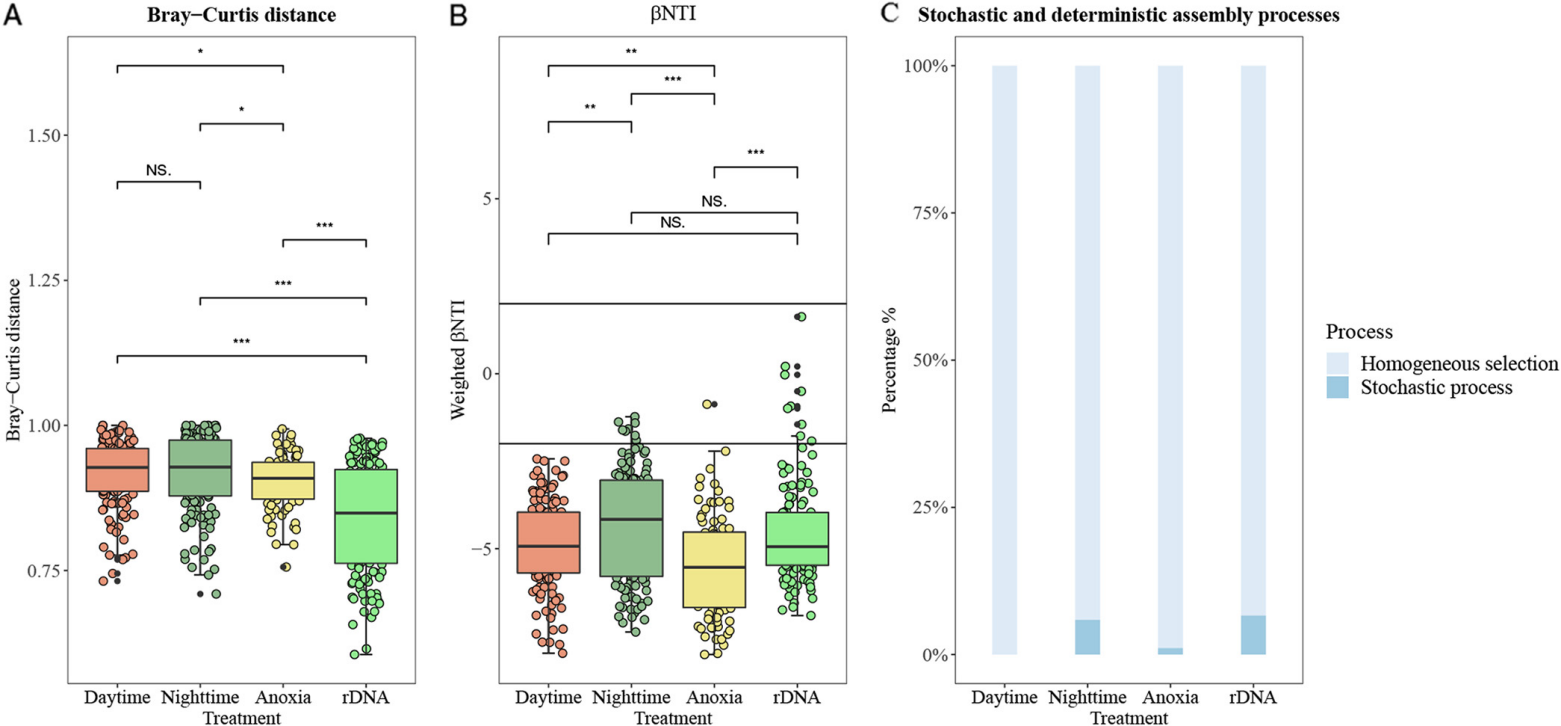
Community assembly processes of total and active phycospheric communities under three conditions: the Bray-Curtis distances (A), the β-NTIs (B), and the proportions of stochastic and deterministic assembly processes of the phycospheric communities (C). The line inside each box represents the median, while the whiskers represent the lowest and highest values. *P* values from pairwise comparisons are shown at the top as follows: ***, *P* < 0.001; **, 0.001 ≤ *P* < 0.01; *, 0.01 ≤ *P* < 0.05.

Phycospheric communities from diurnal and anoxic conditions showed different levels of dissimilarities based on Bray-Curtis distances. Specifically, rDNA samples exhibited the greatest range of dissimilarities, whereas rRNA samples from the anoxic condition showed the lowest level of dissimilarities (Fig. 6A). The results suggested that the total phycospheric communities were in general more divergent, whereas active phycospheric communities, especially those under the anoxic condition, were more similar. Analysis of the β nearest-taxon index (βNTI) further revealed a majority (93.4% to 100%) of pairwise βNTI values were below −2 under all conditions (Fig. 6B), suggesting the assembly of the total and active CA communities were primarily governed by homogeneous selection. Particularly for samples under the conditions of daytime and anoxia, the proportions of homogeneous selection were almost 100% (Fig. 6C). In comparison, the proportion of stochastic processes were relatively higher in nighttime samples. These results together revealed differences in assembly mechanisms in total and active phycospheric communities under various conditions in CAs.

## DISCUSSION

### Effect of cyanobacterial turnover on phycospheric community succession.

Our results showed that the types of cyanobacteria and hydrophysicochemical factors together shaped the composition of phycospheric microbial communities, with cyanobacterial composition demonstrating greater influence. The close relationship between phycospheric bacteria and cyanobacteria was regarded to be in part dependent on metabolic and functional complementarity (42, 43). Here, different cyanobacteria were associated with distinct bacterial lineages. For example, *Cytophagales* and *Burkholderiales* showed negative associations with *Microcystis* populations, yet were positively associated with *Pseudanabaenales.* Indeed, many members of *Cytophaga* exhibit strong algicidal activities (44–47), and some were capable of lysing *Microcystis* cells and utilizing large macromolecules from its debris (48, 49). *Burkholderiales* were also an abundant cyanobacterium-associated group and were often predicted or experimentally verified to be microcystin degraders (50). These results, together with those from many other studies, suggest succession of cyanobacterial composition could drive the turnover of phycospheric communities in the aquatic ecosystem (51).

The close relationship between cyanobacteria and phycospheric bacterial communities was also evidenced at the level of *Microcystis* populations. This study and many others have found diverse *Microcystis* populations in freshwater ecosystems. However, much less attention was paid on how phycospheric communities varied with cyanobacterial genotypes. Li et al. reported colonies of six *Microcystis* species harbored different phycospheric bacterial communities based on metagenomic sequencing (52). A recent study by Pérez-Carrascal et al. assessed genomic diversities of 109 individual *Microcystis* colonies and also found that each *Microcystis* genotype harbored a different microbiome composition (53). The significant temporal partitioning of *Microcystis* populations observed in this study, together with the synchronous turnover of phycospheric communities, further confirmed the importance of tracing *Microcystis* population dynamics rather than considering *Microcystis* as a whole in aquatic ecological studies.

### The relationship between abundances and activities of microbial taxa.

It is generally believed that temporary transcriptive activities of microorganisms were more susceptive to short-term environmental conditions given the much shorter lifetime of RNA than DNA molecules. However, this study revealed that relative activities of individual microbial taxa were predominantly determined by their rDNA-based abundances, with only a minor impact from external conditions. In particular, relative activities of individual taxa were negatively correlated with rDNA-based abundances, which corresponds to higher activities of low-abundance taxa yet lower activities of high-abundance taxa. This finding further challenges the common belief that rarity was typically associated with dormancy, whereas abundant taxa were prone to be metabolically active as major players of ecosystem functions (54). During the past decade, the roles of low-abundance taxa in microbial communities have received increased attention as they compose the majority of microbial diversity (37, 55–58), but the metabolic states and controlling factors of low-abundance taxa were much less understood (59). Emerging yet very limited evidence has shown that low-abundance taxa may nonetheless exhibit disproportionate activities (36, 57). A first explanation of decreased relative activity with abundance may be associated with different growth stages of bacteria. For rare or dormant bacteria, initial growth or resuscitation could be triggered by the availability of specific nutrients or other suitable conditions, followed by rapid and continued bacterial growth and high metabolic activities (59, 60). However, at the later stage of proliferation, a density-dependent resource constraint could be enhanced, hence elevating intraspecific competition and resulting in decreased rates of bacterial growth and metabolism at high abundance levels (36, 61). This was particularly exemplified in this study in that for the several dominant populations, relative activities decreased during periods of high abundance. Specifically, the relative rDNA-based abundances of related taxa spanned up to 2 orders of magnitude across the sampling period (see Fig. S5 in the supplemental material), further supporting that abundances may to some extent reflect the growth stages of these taxa.

In addition, an increasing array of studies have suggested low-abundance taxa could serve important ecosystem functions, thus having high activities despite their low abundances. Known examples include ammonia-oxidizing bacteria and archaea in soils (62, 63), diazotrophs in seawater (64), and sulfur-oxidizing bacteria in acid mine drainage (65). In this study, low-abundance orders such as *Bacteroidales* and *Myxococcales* showed high relative activities at most time points (Fig. 5). The former group contains many active degraders under anoxic conditions, whereas the latter is known to be specialized in particulate organic matter decomposition (66, 67). Hence, both groups may play important roles in turnover of organic materials within CAs. Further investigation of these relatively rare yet highly active bacterial lineages may facilitate better understanding of keystone species and related ecological processes in CAs. Last but not least, studies have also reported trade-off effects for some bacterial species between reproduction and defense mechanisms (68, 69). It is generally known that bacteria with high abundances face increasing pressure from predation and viral lysis. Indeed, it was found that increasing resources may be devoted to defense strategies at high abundances, which could result in reduced growth rates (70). Although the present study lacks evidence of predation or viral lysis, it pointed out that future research could further explore the association of such effects with abundance distributions at the community level. It should be noted that assessment of relative activities in the present study were solely based on 16S rRNA- and rDNA-based abundances. rRNAs are essential molecules required for protein biosynthesis; hence, rRNA abundances are linked to translational activities and commonly used to reflect the overall metabolic states of microorganisms in ecological studies (71, 72). However, transcriptional changes of specific metabolic functions cannot be assessed through this approach, and transcriptomic profiling would provide better resolution on such regulatory responses, especially upon short-term perturbation.

Results from this study also revealed additional factors impacting microbial activities within CAs. First, we found that *Microcystis* exhibited substantially higher relative activities than nearly all other taxa. Since *Microcystis* was the absolute dominant cyanobacterium in all samples taken, the central metabolic processes of *Microcystis* sustained colonization and thriving of all phycospheric communities. Hence, niche partitioning between host cyanobacteria and nonhost communities may in part contribute to differentiated overall levels of relative activities between *Microcystis* and non-*Microcystis*. Second, environmental factors accounted for an additional yet much smaller fraction of variance in relative activities of phycospheric communities. Here, DO, inorganic N, and DIP were the factors with the highest overall impact, which was consistent with findings of many other studies: e.g., oxygen level being a key regulatory factor of aquatic ecosystem processes and the presence of competition between cyanobacteria and phycospheric bacteria for nitrogen and phosphorus sources (73–75). However, no environmental factors could explain the residuals of relative activities of *Microcystis* with the rDNA abundances. This suggests that environmental factors mainly affected the relative activities of *Microcystis* through affecting the dynamics of its rDNA-based abundances, whereas for phycospheric bacterial communities, environmental factors affected their relative abundances not only directly, but also indirectly both through regulating their rDNA-based abundance distribution and through regulating cyanobacterial succession, which further shaped the composition of phycospheric bacterial communities.

### Responses of cyanobacterial activities to diurnal changes and anoxia.

Diurnal change is a key regulator of metabolic activities, especially for photosynthetic autotrophic organisms. Particularly for cyanobacterial aggregates, formation of anaerobic spots under dark conditions may further affect the physiology and metabolism of both the cyanobacteria and phycospheric bacteria. For *Nostocales* and *Chroococcales*, lowered activities during nighttime or under anoxia may relate to blocked photosynthetic electron transport and shifted metabolism toward DNA synthesis, repair, and other activities (20, 21, 76–78). Nonetheless, *Chroococcales* exhibited relatively stable activities throughout the sampling period and maintained activity (with an rRNA/rDNA ratio of >1) under anoxia in most time points, suggesting that *Microcystis* may harbor unique adaptive strategies toward darkness and hypoxia stress. Studies have found *Microcystis* cells that sank to the anoxic sediment-water interface in the past year could survive and maintain a vegetative state through to the next summer (79, 80). Chen et al. reported that under anoxia, *Microcystis* could stay alive for over a week and continuously release organic carbon compounds, algal toxins, and other substances (29). These results together highlight the ecological importance of further studying the adaptive mechanisms of *Microcystis* under dark and anoxic conditions (81–83).

### Assembly mechanisms of active CA communities.

This study found that assembly of CA-associated communities was predominantly governed by homogeneous selection. This is different from microbial communities associated with many other habitat types: e.g., those in soil and sediment, whose assembly was usually dominated by stochastic processes (84–86). Microbes in CAs are typical host-associated communities, where host filtration provides the most important selection effect (87, 88). Known mechanisms involve chemotaxis attraction toward phytoplankton exudates and exchange of molecules, including carbon, iron, organic sulfur, and vitamins, as well as a range of other syntrophic or symbiotic interactions (42). Consistently, it has been reported that microbial communities attached to phytoplankton showed less of an impact of the stochastic assembly process than free-living microbes (89). Hence, our results and others’ together support the idea that conditions within CAs could provide strong selective power toward colonization of specific microbial lineages and exert a homogenizing effect on phylogenetic turnover.

In particular, active microbial communities during daytime showed the highest level of homogeneous selection, followed by those under an anoxic condition. This may be associated with the levels of stress created by the two types of conditions. Specifically, for both obligate and facultative aerobes using oxygen as the terminal electron acceptor, anoxia could stall the electron transfer reactions and interrupt the ATP generation process. Heterotrophic aerobes may respond to the energy stress by entering a growth-arrested state, rewiring essential anabolic pathways, especially for those that require oxygen, or producing redox-active molecules that facilitate electron shuttling anaerobically (90, 91). Hence, anoxia may specifically select for microbial lineages that are better adapted in the absence of oxygen, resulting in significantly lower βNTIs (β-nearest taxon indices), indicating a strong homogenizing effect on phylogenetic diversity.

The daylight condition could also induce a substantial stress response associated with UV radiation, especially considering that daytime samples were collected in the afternoon from surface water. High UV exposure damages essential molecules in cells, such as proteins, pigments, and nucleic acids, further interfering with processes of photosynthesis, nutrient uptake, motility, and DNA replication and transcription (92). In addition, active photosynthesis by cyanobacteria during daytime creates highly oxygenated centers within CAs (26), leading to overproduction and toxic accumulation of reactive oxygen species, which could oxidize a variety of macromolecules and affect cell survival (93–95). Hence, combined effects of UV radiation and oxidative stress may together affect the metabolic states of microbial communities and exert selective effect on activities of specific microbial lineages.

Collectively, these results suggest that host filtration combined with an external stress condition together affect the physiology and assembly mechanisms of active microbial communities within CAs. Nonetheless, transcriptomic analyses are needed to further reveal the response mechanisms to different types of stress and conditions by specific microbial lineages.

## MATERIALS AND METHODS

### Study site and sampling scheme.

The study site was Shazhu of east Lake Taihu, China (31°406′N, 120°232′E). Sampling of CAs and lake water was carried out from May to November in 2019, and a total of 17 samplings were carried out. For each sampling, daytime and nighttime CA samples were taken at 3:00 p.m. and 3:00 a.m., respectively. In brief, water samples were collected from 0.5 m below the surface for water quality analysis; CAs were collected with a 200-mesh (200-μm-pore) plankton net, followed by washing with deionized water to further remove free-living microorganisms. Parts of CA samples were mixed with RNAlater solution for RNA protection (Thermo Fisher Scientific, USA). Water and CA samples were immediately transported on ice to the laboratory for further processing.

### Hydrophysicochemical analyses.

WT, pH, turbidity (Tur), EC, and DO were measured using a YSI 6600-V2 multiparameter sonde (YSI, Yellow Springs, OH, USA). Lake water was filtered through 0.22-μm-pore polycarbonate filter membranes, and the filtrate was used for hydrochemical analyses in the laboratory and for the incubation experiment. DIP and DIN, including nitrate-N, nitrite-N, and ammonia-N, were determined with a UV spectrophotometer (METASH, Shanghai, China) according to standard methods (96).

### Anaerobic incubation.

Nighttime CAs were added into 100-mL serum bottles with sterile lake water to a final chlorophyll concentration of about 0.5 mg/L. The bottles were flushed with nitrogen continuously with a gas needle before and after CAs were added for about 10 min to remove oxygen. Bottles were sealed with butyl rubber stoppers and aluminum caps and incubated at 25°C in dark for 12 h. Collected CA samples were immediately mixed with RNAlater solution for protection of RNA.

### Nucleic acid extraction and amplicon sequencing.

For DNA extraction, washed CA samples were further filtered through a 0.2-μm-pore membrane filter, which was then frozen at −80°C in a refrigerator until DNA extraction. DNA was extracted using the E.Z.N.A. water DNA minikit (Omega Bio-Tek, USA) according to the manufacturer’s instructions. The quality and quantity of extracted DNA were assessed with a NanoDrop spectrophotometer (Merinton Instrument, USA).

CA samples mixed with RNAlater solution were used for RNA extraction. CA samples were first filtered through a 0.2-μm-pore membrane filter, which was then used for RNA extraction with the RNeasy PowerWater kit (Qiagen, Germany) according to the manufacturer’s instructions. The extracted RNA was treated with the Turbo DNA-free kit (Thermo Fisher Scientific, USA) for DNA removal. The obtained RNA was assessed with a Qubit RNA integrity and quality assay kit (Thermo Fisher Scientific, USA) on a Qubit 4 fluorometer (Thermo Fisher Scientific, USA). RNAs were further converted to single-stranded cDNA with the QuantiTect reverse transcription kit (Qiagen, Germany). The V4 region of the 16S rRNA gene was amplified for all DNA and cDNA samples with the primers 515F (5‘-GTGCCAGCMGCCGCGGTAA-3’) and 806R (5‘-GGACTACHVGGGTWTCTAAT-3’) (97). The NEBNext Ultra II DNA library prep kit for Illumina (New England Biolabs, USA) was used for library construction according to the manufacturer’s instructions. Sequencing was performed at the Guangdong Magigene Biotechnology Co., Ltd. (Guangzhou, China), on the Illumina Hiseq 2500 platform, and all samples were rarefied to the same number of reads: 21,160.

### Data processing and statistical analyses.

Amplicon sequence profiles were processed using the QIIME 2 pipeline (QIIME 2 v.2020.8.0) (98). In brief, raw sequences were demultiplexed and quality controlled using the q2-demux plugin. DADA2 was used for denoising, deredundancy, and clustering of amplicon sequence variants (ASVs) (99). All ASVs were aligned with mafft (via q2-alignment), and phylogeny was constructed with fasttree2 (via q2-phylogeny) (100, 101). Taxonomic assignment was through the Bayes taxonomy classifier (q2-feature-classifier classify-sklearn naive) against the Greengenes 13_8 99% operational taxonomic unit (OTU) reference sequences (102, 103). ASVs matched to mitochondria and chloroplasts were removed.

Statistical analyses were mainly performed in R 4.1.3 and R 3.6.3 with “vegan” package 2.6.2 unless otherwise specified (104). The “ape” package was used for PCoA. Differentially distributed ASVs among different months were identified with DESeq2 (105). It should be noted that microbial communities generally have rank-abundance curves with long tails and hence large fractions of low-abundance taxa. These low-abundance taxa were heavily affected by the effect of random sampling. Inclusion of these zero values could also lead to problematic estimations of 16S rRNA/rDNA ratios, especially for those with zero-rDNA values, hence masking the real relationship between relative activities and rDNA-based abundances. Therefore, the zero-value data points were removed from our regression analyses.

Community assembly processes were evaluated with the null model analysis according to the methods described by Stegen et al. (106–108) and with the Picante package (106). Briefly, the β mean nearest-taxon distance (βMNTD)—the abundance-weighted average phylogenetic distance between closest relatives in each pair of communities—was first calculated for each pairwise sample comparison. The null communities were next generated by randomizing the community structure 999 times, and a null distribution of βMNTD was generated by calculating βMNTDs for each randomization. The β nearest-taxon index (βNTI) is the difference of the observed βMNTD from the mean of the βMNTD null distribution normalized by the standard deviation. A βNTI of less than −2 indicates significantly lower phylogenetic turnover than expected and was used to infer a homogeneous selection process; all other βNTIs were between −2 and 2 in this study and indicate a stochastic process for the specific pairwise comparison.

### Data availability.

Data associated with this study were deposited at the NODE (the National Omics Data Encyclopedia [https://www.biosino.org/node]) database under project no. OEP003624.

## References

[1] Paerl HW, Gardner WS, McCarthy MJ, Peierls BL, Wilhelm SW. 2014. Algal blooms: noteworthy nitrogen. Science 346:175. doi:10.1126/science.346.6206.175-a. 25301607

[2] Paerl HW, Huisman J. 2008. Blooms like it hot. Science 320:57–58. doi:10.1126/science.1155398. 18388279

[3] Havens KE. 2008. Cyanobacteria blooms: effects on aquatic ecosystems, p 733–747. *In* Hudnell HK (ed), Cyanobacterial harmful algal blooms: state of the science and research needs. Springer, New York, NY. 10.1007/978-0-387-75865-7_33 18461790

[4] Xu H, Jiang H, Yu G, Yang L. 2014. Towards understanding the role of extracellular polymeric substances in cyanobacterial *Microcystis* aggregation and mucilaginous bloom formation. Chemosphere 117:815–822. doi:10.1016/j.chemosphere.2014.10.061. 25465953

[5] Xu H, Yu G, Jiang H. 2013. Investigation on extracellular polymeric substances from mucilaginous cyanobacterial blooms in eutrophic freshwater lakes. Chemosphere 93:75–81. doi:10.1016/j.chemosphere.2013.04.077. 23726883

[6] Deng J, Chen X, Huang Y. 2020. Biogeochemical cycling processes associated with cyanobacterial aggregates. Acta Microbiol Sinica 60:1922–1940.

[7] Grant MAA, Kazamia E, Cicuta P, Smith AG. 2014. Direct exchange of vitamin B12 is demonstrated by modelling the growth dynamics of algal-bacterial cocultures. ISME J 8:1418–1427. doi:10.1038/ismej.2014.9. 24522262 PMC4069406

[8] Li Y, Hongyi W, Komatsu M, Ishibashi K, Jinsan L, Ito T, Yoshikawa T, Maeda H. 2012. Isolation and characterization of bacterial isolates algicidal against a harmful bloom-forming cyanobacterium *Microcystis* aeruginosa. Biocontrol Sci 17:107–114. doi:10.4265/bio.17.107. 23007101

[9] Mayali X, Azam F. 2004. Algicidal bacteria in the sea and their impact on algal blooms. J Eukaryot Microbiol 51:139–144. doi:10.1111/j.1550-7408.2004.tb00538.x. 15134248

[10] Heal KR, Qin W, Ribalet F, Bertagnolli AD, Coyote-Maestas W, Hmelo LR, Moffett JW, Devol AH, Armbrust EV, Stahl DA, Ingalls AE. 2017. Two distinct pools of B_12_ analogs reveal community interdependencies in the ocean. Proc Natl Acad Sci USA 114:364–369. doi:10.1073/pnas.1608462114. 28028206 PMC5240700

[11] Xie M, Ren M, Yang C, Yi H, Li Z, Li T, Zhao J. 2016. Metagenomic analysis reveals symbiotic relationship among bacteria in *Microcystis*-dominated community. Front Microbiol 7:56. doi:10.3389/fmicb.2016.00056. 26870018 PMC4735357

[12] Kazamia E, Czesnick H, Nguyen TT, Croft MT, Sherwood E, Sasso S, Hodson SJ, Warren MJ, Smith AG. 2012. Mutualistic interactions between vitamin B_12_-dependent algae and heterotrophic bacteria exhibit regulation. Environ Microbiol 14:1466–1476. doi:10.1111/j.1462-2920.2012.02733.x. 22463064

[13] Hayashi S, Itoh K, Suyama K. 2011. Growth of the cyanobacterium *Synechococcus leopoliensis* CCAP1405/1 on agar media in the presence of heterotrophic bacteria. Microbes Environ 26:120–127. doi:10.1264/jsme2.me10193. 21502741

[14] Amin SA, Green DH, Hart MC, Küpper FC, Sunda WG, Carrano CJ. 2009. Photolysis of iron-siderophore chelates promotes bacterial-algal mutualism. Proc Natl Acad Sci USA 106:17071–17076. doi:10.1073/pnas.0905512106. 19805106 PMC2761308

[15] Zhu C, Zhang J, Guan R, Hale L, Chen N, Li M, Lu Z, Ge Q, Yang Y, Zhou J, Chen T. 2019. Alternate succession of aggregate-forming cyanobacterial genera correlated with their attached bacteria by co-pathways. Sci Total Environ 688:867–879. doi:10.1016/j.scitotenv.2019.06.150. 31255824

[16] Zhu C, Zhang J, Nawaz MZ, Mahboob S, Al-Ghanim KA, Khan IA, Lu Z, Chen T. 2019. Seasonal succession and spatial distribution of bacterial community structure in a eutrophic freshwater lake, Lake Taihu. Sci Total Environ 669:29–40. doi:10.1016/j.scitotenv.2019.03.087. 30877958

[17] Latifi A, Ruiz M, Zhang C. 2009. Oxidative stress in cyanobacteria. FEMS Microbiol Rev 33:258–278. doi:10.1111/j.1574-6976.2008.00134.x. 18834454

[18] Morris JJ, Kirkegaard R, Szul MJ, Johnson ZI, Zinser ER. 2008. Facilitation of robust growth of *Prochlorococcus* colonies and dilute liquid cultures by “helper” heterotrophic bacteria. Appl Environ Microbiol 74:4530–4534. doi:10.1128/AEM.02479-07. 18502916 PMC2493173

[19] Diamond S, Rubin BE, Shultzaberger RK, Chen Y, Barber CD, Golden SS. 2017. Redox crisis underlies conditional light-dark lethality in cyanobacterial mutants that lack the circadian regulator, RpaA. Proc Natl Acad Sci USA 114:580–589. doi:10.1073/pnas.1613078114. 28074036 PMC5278464

[20] Welkie DG, Rubin BE, Diamond S, Hood RD, Savage DF, Golden SS. 2019. A hard day's night: cyanobacteria in diel cycles. Trends Microbiol 27:231–242. doi:10.1016/j.tim.2018.11.002. 30527541 PMC6377297

[21] Penn K, Wang J, Fernando SC, Thompson JR. 2014. Secondary metabolite gene expression and interplay of bacterial functions in a tropical freshwater cyanobacterial bloom. ISME J 8:1866–1878. doi:10.1038/ismej.2014.27. 24646695 PMC4139720

[22] Johan W, Rassoulzadegan F, Hagström Å. 1990. Periodic bacterivore activity balances bacterial growth in the marine environment. Limnol Oceanogr 35:313–324. doi:10.4319/lo.1990.35.2.0313.

[23] Fang F, Gao Y, Gan L, He XY, Yang LY. 2018. Effects of different initial pH and irradiance levels on cyanobacterial colonies from Lake Taihu, China. J Appl Phycol 30:1777–1793. doi:10.1007/s10811-018-1394-5.

[24] Bianchi D, Weber TS, Kiko R, Deutsch C. 2018. Global niche of marine anaerobic metabolisms expanded by particle microenvironments. Nat Geosci 11:263–268. doi:10.1038/s41561-018-0081-0.

[25] Klawonn I, Bonaglia S, Brüchert V, Ploug H. 2015. Aerobic and anaerobic nitrogen transformation processes in N_2_-fixing cyanobacterial aggregates. ISME J 9:1456–1466. doi:10.1038/ismej.2014.232. 25575306 PMC4438332

[26] Eichner MJ, Klawonn I, Wilson ST, Littmann S, Whitehouse MJ, Church MJ, Kuypers MM, Karl DM, Ploug H. 2017. Chemical microenvironments and single-cell carbon and nitrogen uptake in field-collected colonies of *Trichodesmium* under different pCO2. ISME J 11:1305–1317. doi:10.1038/ismej.2017.15. 28398346 PMC5437350

[27] Krishnan A, Zhang S, Liu Y, Tadmori KA, Bryant DA, Dismukes CG. 2016. Consequences of ccmR deletion on respiration, fermentation and H_2_ metabolism in cyanobacterium *Synechococcus sp*. PCC 7002. Biotechnol Bioeng 113:1448–1459. doi:10.1002/bit.25913. 26704377

[28] Stockel J, Welsh EA, Liberton M, Kunnvakkam R, Aurora R, Pakrasi HB. 2008. Global transcriptomic analysis of Cyanothece 51142 reveals robust diurnal oscillation of central metabolic processes. Proc Natl Acad Sci USA 105:6156–6161. doi:10.1073/pnas.0711068105. 18427117 PMC2329701

[29] Chen X, Huang Y, Chen G, Li P, Shen Y, Davis TW. 2018. The secretion of organics by living *Microcystis* under the dark/anoxic condition and its enhancing effect on nitrate removal. Chemosphere 196:280–287. doi:10.1016/j.chemosphere.2017.12.197. 29306780

[30] Grim SL, Voorhies AA, Biddanda BA, Jain S, Nold SC, Green R, Dick GJ. 2021. Omics-inferred partitioning and expression of diverse biogeochemical functions in a low-O_2_ cyanobacterial mat community. mSystems 6:e01042-21. doi:10.1128/mSystems.01042-21. 34874776 PMC8651085

[31] Lawson CE, Strachan BJ, Hanson NW, Hahn AS, Hall ER, Rabinowitz B, Mavinic DS, Ramey WD, Hallam SJ. 2015. Rare taxa have potential to make metabolic contributions in enhanced biological phosphorus removal ecosystems. Environ Microbiol 17:4979–4993. doi:10.1111/1462-2920.12875. 25857222

[32] Bergen B, Naumann M, Herlemann DPR, Grawe U, Labrenz M, Jurgens K. 2018. Impact of a major inflow event on the composition and distribution of bacterioplankton communities in the Baltic Sea. Front Mar Sci 5:383. doi:10.3389/fmars.2018.00383.

[33] Schaedler F, Lockwood C, Lueder U, Glombitza C, Kappler A, Schmidt C. 2018. Microbially mediated coupling of Fe and N cycles by nitrate-reducing Fe(II)-oxidizing bacteria in littoral freshwater sediments. Appl Environ Microbiol 84:e02013-17. doi:10.1128/AEM.02013-17. 29101195 PMC5752864

[34] Hunt DE, Lin YJ, Church MJ, Karl DM, Tringe SG, Izzo LK, Johnson ZI. 2013. Relationship between abundance and specific activity of bacterioplankton in open ocean surface waters. Appl Environ Microbiol 79:177–184. doi:10.1128/AEM.02155-12. 23087033 PMC3536108

[35] Barreto MM, Ziegler M, Venn A, Tambutte E, Zoccola D, Tambutte S, Allemand D, Antony CP, Voolstra CR, Aranda M. 2021. Effects of ocean acidification on resident and active microbial communities of *Stylophora pistillata*. Front Microbiol 12:707674. doi:10.3389/fmicb.2021.707674. 34899619 PMC8656159

[36] Campbell BJ, Yu LY, Heidelberg JF, Kirchman DL. 2011. Activity of abundant and rare bacteria in a coastal ocean. Proc Natl Acad Sci USA 108:12776–12781. doi:10.1073/pnas.1101405108. 21768380 PMC3150899

[37] Jones SE, Lennon JT. 2010. Dormancy contributes to the maintenance of microbial diversity. Proc Natl Acad Sci USA 107:5881–5886. doi:10.1073/pnas.0912765107. 20231463 PMC2851880

[38] Lee MD, Walworth NG, McParland EL, Fu FX, Mincer TJ, Levine NM, Hutchins DA, Webb EA. 2017. The *Trichodesmium* consortium: conserved heterotrophic co-occurrence and genomic signatures of potential interactions. ISME J 11:1813–1824. doi:10.1038/ismej.2017.49. 28440800 PMC5520037

[39] Shi L, Huang Y, Zhang M, Yu Y, Lu Y, Kong F. 2017. Bacterial community dynamics and functional variation during the long-term decomposition of cyanobacterial blooms in-vitro. Sci Total Environ 598:77–86. doi:10.1016/j.scitotenv.2017.04.115. 28437774

[40] Campbell BJ, Kirchman DL. 2013. Bacterial diversity, community structure and potential growth rates along an estuarine salinity gradient. ISME J 7:210–220. doi:10.1038/ismej.2012.93. 22895159 PMC3526181

[41] Gaidos E, Rusch A, Ilardo M. 2011. Ribosomal tag pyrosequencing of DNA and RNA from benthic coral reef microbiota: community spatial structure, rare members and nitrogen-cycling guilds. Environ Microbiol 13:1138–1152. doi:10.1111/j.1462-2920.2010.02392.x. 21176054

[42] Seymour JR, Amin SA, Raina JB, Stocker R. 2017. Zooming in on the phycosphere: the ecological interface for phytoplankton-bacteria relationships. Nat Microbiol 2:17065. doi:10.1038/nmicrobiol.2017.65. 28555622

[43] Zheng Q, Wang Y, Lu JY, Lin WX, Chen F, Jiao NZ. 2020. Metagenomic and metaproteomic insights into photoautotrophic and heterotrophic interactions in a *Synechococcus* culture. mBio 11:e03261-19. doi:10.1128/mBio.03261-19. 32071270 PMC7029141

[44] Roth PB, Twiner MJ, Mikulski CM, Barnhorst AB, Doucette GJ. 2008. Comparative analysis of two algicidal bacteria active against the red tide dinoflagellate *Karenia brevis*. Harmful Algae 7:682–691. doi:10.1016/j.hal.2008.02.002.

[45] Mayali X, Doucette GJ. 2002. Microbial community interactions and population dynamics of an algicidal bacterium active against *Karenia brevis (Dinophyceae)*. Harmful Algae 1:277–293. doi:10.1016/S1568-9883(02)00032-X.

[46] Gerphagnon M, Macarthur DJ, Latour D, Gachon CMM, Van Ogtrop F, Gleason FH, Sime-Ngando T. 2015. Microbial players involved in the decline of filamentous and colonial cyanobacterial blooms with a focus on fungal parasitism. Environ Microbiol 17:2573–2587. doi:10.1111/1462-2920.12860. 25818470

[47] Nagasaki K, Yamaguchi M, Imai I. 2000. Algicidal activity of a killer bacterium against the harmful red tide dinoflagellate Heterocapsa circularisquama isolated from Ago Bay, Japan. Nippon Suisan Gakkaishi 66:666–673. doi:10.2331/suisan.66.666.

[48] Guedes IA, Rachid CTCC, Rangel LM, Silva LHS, Bisch PM, Azevedo SMFO, Pacheco ABF. 2018. Close link between harmful cyanobacterial dominance and associated bacterioplankton in a tropical eutrophic reservoir. Front Microbiol 9:424. doi:10.3389/fmicb.2018.00424. 29593677 PMC5857610

[49] Maruyama T, Kato K, Yokoyama A, Tanaka T, Hiraishi A, Park HD. 2003. Dynamics of microcystin-degrading bacteria in mucilage of *Microcystis*. Microb Ecol 46:279–288. doi:10.1007/s00248-002-3007-7. 14708752

[50] Mou XZ, Lu XX, Jacob J, Sun SL, Heath R. 2013. Metagenomic identification of bacterioplankton taxa and pathways involved in microcystin degradation in Lake Erie. PLoS One 8:e61890. doi:10.1371/journal.pone.0061890. 23637924 PMC3634838

[51] Bagatini IL, Eiler A, Bertilsson S, Klaveness D, Tessarolli LP, Vieira AAH. 2014. Host-specificity and dynamics in bacterial communities associated with bloom-forming freshwater phytoplankton. PLoS One 9:e85950. doi:10.1371/journal.pone.0085950. 24465807 PMC3896425

[52] Li Q, Lin F, Yang C, Wang J, Lin Y, Shen M, Park MS, Li T, Zhao J. 2018. A large-scale comparative metagenomic study reveals the functional interactions in six bloom-forming *Microcystis*-epibiont communities. Front Microbiol 9:746. doi:10.3389/fmicb.2018.00746. 29731741 PMC5919953

[53] Perez-Carrascal OM, Tromas N, Terrat Y, Moreno E, Giani A, Correa Braga Marques L, Fortin N, Shapiro BJ. 2021. Single-colony sequencing reveals microbe-by-microbiome phylosymbiosis between the cyanobacterium *Microcystis* and its associated bacteria. Microbiome 9:194. doi:10.1186/s40168-021-01140-8. 34579777 PMC8477515

[54] Pedrós-Alió C. 2006. Marine microbial diversity: can it be determined? Trends Microbiol 14:257–263. doi:10.1016/j.tim.2006.04.007. 16679014

[55] Elshahed MS, Youssef NH, Spain AM, Sheik C, Najar FZ, Sukharnikov LO, Roe BA, Davis JP, Schloss PD, Bailey VL, Krumholz LR. 2008. Novelty and uniqueness patterns of rare members of the soil biosphere. Appl Environ Microbiol 74:5422–5428. doi:10.1128/AEM.00410-08. 18606799 PMC2546616

[56] Sogin ML, Morrison HG, Huber JA, Welch DM, Huse SM, Neal PR, Arrieta JM, Herndl GJ. 2006. Microbial diversity in the deep sea and the underexplored “rare biosphere”. Proc Natl Acad Sci USA 103:12115–12120. doi:10.1073/pnas.0605127103. 16880384 PMC1524930

[57] Lennon JT, Jones SE. 2011. Microbial seed banks: the ecological and evolutionary implications of dormancy. Nat Rev Microbiol 9:119–130. doi:10.1038/nrmicro2504. 21233850

[58] Aanderud ZT, Jones SE, Fierer N, Lennon JT. 2015. Resuscitation of the rare biosphere contributes to pulses of ecosystem activity. Front Microbiol 6:24. doi:10.3389/fmicb.2015.00024. 25688238 PMC4311709

[59] Shade A, Jones SE, Caporaso JG, Handelsman J, Knight R, Fierer N, Gilbert JA. 2014. Conditionally rare taxa disproportionately contribute to temporal changes in microbial diversity. mBio 5:e01371-14. doi:10.1128/mBio.01371-14. 25028427 PMC4161262

[60] Klappenbach JA, Dunbar JM, Schmidt TM. 2000. rRNA operon copy number reflects ecological strategies of bacteria. Appl Environ Microbiol 66:1328–1333. doi:10.1128/AEM.66.4.1328-1333.2000. 10742207 PMC91988

[61] Deutscher MP. 2003. Degradation of stable RNA in bacteria. J Biol Chem 278:45041–45044. doi:10.1074/jbc.R300031200. 12941949

[62] Le Roux X, Bouskill NJ, Niboyet A, Barthes L, Dijkstra P, Field CB, Hungate BA, Lerondelle C, Pommier T, Tang JY, Terada A, Tourna M, Poly F. 2016. Predicting the responses of soil nitrite-oxidizers to multi-factorial global change: a trait-based approach. Front Microbiol 7:628. doi:10.3389/fmicb.2016.00628. 27242680 PMC4868854

[63] Leininger S, Urich T, Schloter M, Schwark L, Qi J, Nicol GW, Prosser JI, Schuster SC, Schleper C. 2006. Archaea predominate among ammonia-oxidizing prokaryotes in soils. Nature 442:806–809. doi:10.1038/nature04983. 16915287

[64] LaRoche J, Breitbarth E. 2005. Importance of the diazotrophs as a source of new nitrogen in the ocean. J Sea Res 53:67–91. doi:10.1016/j.seares.2004.05.005.

[65] Hua ZS, Han YJ, Chen LX, Liu J, Hu M, Li SJ, Kuang JL, Chain PSG, Huang LN, Shu WS. 2015. Ecological roles of dominant and rare prokaryotes in acid mine drainage revealed by metagenomics and metatranscriptomics. ISME J 9:1280–1294. doi:10.1038/ismej.2014.212. 25361395 PMC4438317

[66] Martinez-Fernandez G, Jiao JZ, Padmanabha J, Denman SE, McSweeney CS. 2020. Seasonal and nutrient supplement responses in rumen microbiota structure and metabolites of tropical rangeland cattle. Microorganisms 8:1550. doi:10.3390/microorganisms8101550. 33049981 PMC7600044

[67] Wang WJ, Liu AR, Fu WT, Peng DL, Wang G, Ji J, Jin C, Guan CF. 2022. Tobacco-associated with *Methylophilus sp*. FP-6 enhances phytoremediation of benzophenone-3 through regulating soil microbial community, increasing photosynthetic capacity and maintaining redox homeostasis of plant. J Hazard Mater 431:128588. doi:10.1016/j.jhazmat.2022.128588. 35248957

[68] Lauro FM, McDougald D, Thomas T, Williams TJ, Egan S, Rice S, DeMaere MZ, Ting L, Ertan H, Johnson J, Ferriera S, Lapidus A, Anderson I, Kyrpides N, Munk AC, Detter C, Han CS, Brown MV, Robb FT, Kjelleberg S, Cavicchioli R. 2009. The genomic basis of trophic strategy in marine bacteria. Proc Natl Acad Sci USA 106:15527–15533. doi:10.1073/pnas.0903507106. 19805210 PMC2739866

[69] Yooseph S, Nealson KH, Rusch DB, McCrow JP, Dupont CL, Kim M, Johnson J, Montgomery R, Ferriera S, Beeson K, Williamson SJ, Tovchigrechko A, Allen AE, Zeigler LA, Sutton G, Eisenstadt E, Rogers YH, Friedman R, Frazier M, Venter JC. 2010. Genomic and functional adaptation in surface ocean planktonic prokaryotes. Nature 468:60–66. doi:10.1038/nature09530. 21048761

[70] Winter C, Bouvier T, Weinbauer MG, Thingstad TF. 2010. Trade-offs between competition and defense specialists among unicellular planktonic organisms: the “Killing the winner” hypothesis revisited. Microbiol Mol Biol Rev 74:42–57. doi:10.1128/MMBR.00034-09. 20197498 PMC2832346

[71] Cutter MR, Stroot PG. 2008. Determination of specific growth rate by measurement of specific rate of ribosome synthesis in growing and nongrowing cultures of *Acinetobacter calcoaceticus*. Appl Environ Microbiol 74:901–903. doi:10.1128/AEM.01899-07. 18083876 PMC2227727

[72] Perez-Osorio AC, Williamson KS, Franklin MJ. 2010. Heterogeneous *rpoS* and *rhlR* mRNA levels and 16S rRNA/rDNA (rRNA gene) ratios within *Pseudomonas aeruginosa* biofilms, sampled by laser capture microdissection. J Bacteriol 192:2991–3000. doi:10.1128/JB.01598-09. 20348255 PMC2901698

[73] Deng WC, Wang SL, Wan XH, Zheng ZZ, Jiao NAZ, Kao SJ, Moore JK, Zhang Y. 2021. Potential competition between marine heterotrophic prokaryotes and autotrophic picoplankton for nitrogen substrates. Limnol Oceanogr 66:3338–3355. doi:10.1002/lno.11883.

[74] Brauer VS, Stomp M, Bouvier T, Fouilland E, Leboulanger C, Confurius-Guns V, Weissing FJ, Stal LJ, Huisman J. 2015. Competition and facilitation between the marine nitrogen-fixing cyanobacterium Cyanothece and its associated bacterial community. Front Microbiol 5:795. doi:10.3389/fmicb.2014.00795. 25642224 PMC4294207

[75] Cunha DGF, Lima VFD, Neri AM, Marafao GA, Miwa ACP, Calijuri MD, Bendassoli JA, Tromboni F, Maranger R. 2017. Uptake rates of ammonium and nitrate by phytoplankton communities in two eutrophic tropical reservoirs. Int Rev Hydrobiol 102:125–134. doi:10.1002/iroh.201701900.

[76] Lepp PW, Schmidt TM. 1998. Nucleic acid content of *Synechococcus spp*. during growth in continuous light and light/dark cycles. Arch Microbiol 170:201–207. doi:10.1007/s002030050634. 9683660

[77] Watanabe S, Ohbayashi R, Shiwa Y, Noda A, Kanesaki Y, Chibazakura T, Yoshikawa H. 2012. Light-dependent and asynchronous replication of cyanobacterial multi-copy chromosomes. Mol Microbiol 83:856–865. doi:10.1111/j.1365-2958.2012.07971.x. 22403820

[78] Ohbayashi R, Watanabe S, Kanesaki Y, Narikawa R, Chibazakura T, Ikeuchi M, Yoshikawa H. 2013. DNA replication depends on photosynthetic electron transport in cyanobacteria. FEMS Microbiol Lett 344:138–144. doi:10.1111/1574-6968.12166. 23621483

[79] Wu Y-F, Xing P, Liu S, Wu Q. 2019. Enhanced microbial interactions and deterministic successions during anoxic decomposition of *Microcystis* biomass in lake sediment. Front Microbiol 10:2474. doi:10.3389/fmicb.2019.02474. 31736913 PMC6831559

[80] Delphine L, Odile S, Marie-José S, Hervé G. 2004. Dynamics and metabolic activity of the benthic cyanobacterium *Microcystis aeruginosa* in the Grangent reservoir (France). J Plankton Res 26:719–726. doi:10.1093/plankt/fbh075.

[81] Vaulot D, Marie D, Olson R, Chisholm S. 1995. Growth of Prochlorococcus, a photosynthetic prokaryote, in the equatorial Pacific Ocean. Science 268:1480–1482. doi:10.1126/science.268.5216.1480. 17843668

[82] Gilbert JA, Field D, Swift P, Thomas S, Cummings D, Temperton B, Weynberg K, Huse S, Hughes M, Joint I, Somerfield PJ, Mühling M. 2010. The taxonomic and functional diversity of microbes at a temperate coastal site: a ‘multi-omic' study of seasonal and diel temporal variation. PLoS One 5:e15545. doi:10.1371/journal.pone.0015545. 21124740 PMC2993967

[83] Ottesen EA, Young CR, Eppley JM, Ryan JP, Chavez FP, Scholin CA, DeLong EF. 2013. Pattern and synchrony of gene expression among sympatric marine microbial populations. Proc Natl Acad Sci USA 110:E488–E497. doi:10.1073/pnas.1222099110. 23345438 PMC3568374

[84] Hou L, Hu A, Chen S, Zhang K, Orlić S, Rashid A, Yu CP. 2019. Deciphering the assembly processes of the key ecological assemblages of microbial communities in thirteen full-scale wastewater treatment plants. Microbes Environ 34:169–179. doi:10.1264/jsme2.ME18107. 30996148 PMC6594736

[85] Logares R, Tesson SV, Canback B, Pontarp M, Hedlund K, Rengefors K. 2018. Contrasting prevalence of selection and drift in the community structuring of bacteria and microbial eukaryotes. Environ Microbiol 20:2231–2240. doi:10.1111/1462-2920.14265. 29727053

[86] Vass M, Szekely AJ, Lindstrom ES, Langenheder S. 2020. Using null models to compare bacterial and microeukaryotic metacommunity assembly under shifting environmental conditions. Sci Rep 10:2455. doi:10.1038/s41598-020-59182-1. 32051469 PMC7016149

[87] Valyi K, Mardhiah U, Rillig MC, Hempel S. 2016. Community assembly and coexistence in communities of arbuscular mycorrhizal fungi. ISME J 10:2341–2351. doi:10.1038/ismej.2016.46. 27093046 PMC5030697

[88] Robinson CJ, Bohannan BJM, Young VB. 2010. From structure to function: the ecology of host-associated microbial communities. Microbiol Mol Biol Rev 74:453–476. doi:10.1128/MMBR.00014-10. 20805407 PMC2937523

[89] Zhou J, Chen GF, Ying KZ, Jin H, Song JT, Cai ZH. 2019. Phycosphere microbial succession patterns and assembly mechanisms in a marine dinoflagellate bloom. Appl Environ Microbiol 85:e00349-19. doi:10.1128/AEM.00349-19. 31126952 PMC6643250

[90] Ciemniecki JA, Newman DK. 2020. The potential for redox-active metabolites to enhance or unlock anaerobic survival metabolisms in aerobes. J Bacteriol 202:e00797-19. doi:10.1128/JB.00797-19. 32071098 PMC7221258

[91] Morris RL, Schmidt TM. 2013. Shallow breathing: bacterial life at low O_2_. Nat Rev Microbiol 11:205–212. doi:10.1038/nrmicro2970. 23411864 PMC3969821

[92] Boelen P, Post AF, Veldhuis MJW, Buma AGJ. 2002. Diel patterns of UVBR-induced DNA damage in picoplankton size fractions from the Gulf of Aqaba, Red Sea. Microb Ecol 44:164–174. doi:10.1007/s00248-002-1002-7. 12060864

[93] Markkanen E. 2017. Not breathing is not an option: how to deal with oxidative DNA damage. DNA Repair (Amst) 59:82–105. doi:10.1016/j.dnarep.2017.09.007. 28963982

[94] Seixas AF, Quendera AP, Sousa JP, Silva AFQ, Arraiano CM, Andrade JM. 2022. Bacterial response to oxidative stress and RNA oxidation. Front Genet 12:821535. doi:10.3389/fgene.2021.821535. 35082839 PMC8784731

[95] Dungan AM, Bulach D, Lin HY, van Oppen MJH, Blackall LL. 2021. Development of a free radical scavenging bacterial consortium to mitigate oxidative stress in cnidarians. Microb Biotechnol 14:2025–2040. doi:10.1111/1751-7915.13877. 34259383 PMC8449677

[96] APHA. 1995. Standard methods for the examination of water and wastewater, 19th ed. American Public Health Association, Washington, DC.

[97] Caporaso JG, Lauber CL, Walters WA, Berg-Lyons D, Huntley J, Fierer N, Owens SM, Betley J, Fraser L, Bauer M, Gormley N, Gilbert JA, Smith G, Knight R. 2012. Ultra-high-throughput microbial community analysis on the Illumina HiSeq and MiSeq platforms. ISME J 6:1621–1624. doi:10.1038/ismej.2012.8. 22402401 PMC3400413

[98] Bolyen E, Rideout JR, Dillon MR, Bokulich NA, Abnet CC, Al-Ghalith GA, Alexander H, Alm EJ, Arumugam M, Asnicar F, Bai Y, Bisanz JE, Bittinger K, Brejnrod A, Brislawn CJ, Brown CT, Callahan BJ, Caraballo-Rodríguez AM, Chase J, Cope EK, Da Silva R, Diener C, Dorrestein PC, Douglas GM, Durall DM, Duvallet C, Edwardson CF, Ernst M, Estaki M, Fouquier J, Gauglitz JM, Gibbons SM, Gibson DL, Gonzalez A, Gorlick K, Guo J, Hillmann B, Holmes S, Holste H, Huttenhower C, Huttley GA, Janssen S, Jarmusch AK, Jiang L, Kaehler BD, Kang KB, Keefe CR, Keim P, Kelley ST, Knights D, et al. 2019. Reproducible, interactive, scalable and extensible microbiome data science using QIIME 2. Nat Biotechnol 37:852–857. doi:10.1038/s41587-019-0209-9. 31341288 PMC7015180

[99] Callahan BJ, McMurdie PJ, Rosen MJ, Han AW, Johnson AJA, Holmes SP. 2016. DADA2: high-resolution sample inference from Illumina amplicon data. Nat Methods 13:581–583. doi:10.1038/nmeth.3869. 27214047 PMC4927377

[100] Price MN, Dehal PS, Arkin AP. 2010. FastTree 2—approximately maximum-likelihood trees for large alignments. PLoS One 5:e9490. doi:10.1371/journal.pone.0009490. 20224823 PMC2835736

[101] Katoh K, Misawa K, Kuma K, Miyata T. 2002. MAFFT: a novel method for rapid multiple sequence alignment based on fast Fourier transform. Nucleic Acids Res 30:3059–3066. doi:10.1093/nar/gkf436. 12136088 PMC135756

[102] Bokulich NA, Kaehler BD, Rideout JR, Dillon M, Bolyen E, Knight R, Huttley GA, Caporaso JG. 2018. Optimizing taxonomic classification of marker-gene amplicon sequences with QIIME 2's q2-feature-classifier plugin. Microbiome 6:90. doi:10.1186/s40168-018-0470-z. 29773078 PMC5956843

[103] McDonald D, Price MN, Goodrich J, Nawrocki EP, DeSantis TZ, Probst A, Andersen GL, Knight R, Hugenholtz P. 2012. An improved Greengenes taxonomy with explicit ranks for ecological and evolutionary analyses of bacteria and archaea. ISME J 6:610–618. doi:10.1038/ismej.2011.139. 22134646 PMC3280142

[104] Dixon P. 2003. VEGAN, a package of R functions for community ecology. J Veg Sci 14:927–930. doi:10.1111/j.1654-1103.2003.tb02228.x.

[105] Love MI, Huber W, Anders S. 2014. Moderated estimation of fold change and dispersion for RNA-seq data with DESeq2. Genome Biol 15:550. doi:10.1186/s13059-014-0550-8. 25516281 PMC4302049

[106] Kembel SW, Cowan PD, Helmus MR, Cornwell WK, Morlon H, Ackerly DD, Blomberg SP, Webb CO. 2010. Picante: R tools for integrating phylogenies and ecology. Bioinformatics 26:1463–1464. doi:10.1093/bioinformatics/btq166. 20395285

[107] Stegen JC, Lin XJ, Konopka AE, Fredrickson JK. 2012. Stochastic and deterministic assembly processes in subsurface microbial communities. ISME J 6:1653–1664. doi:10.1038/ismej.2012.22. 22456445 PMC3498916

[108] Fine PVA, Kembel SW. 2011. Phylogenetic community structure and phylogenetic turnover across space and edaphic gradients in western Amazonian tree communities. Ecography 34:552–565. doi:10.1111/j.1600-0587.2010.06548.x.

