# Supplementary figures and images for: Determinants of Total and Active Microbial Communities Associated with Cyanobacterial Aggregates in a Eutrophic Lake
Source: mSystems. 2023 Mar 16;8(2):e00992-22. doi: 10.1128/msystems.00992-22 (PMC10134853; doi:10.1128/msystems.00992-22)

A

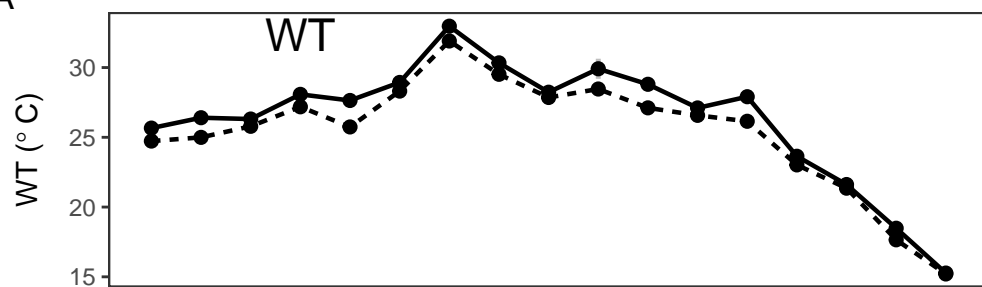

B

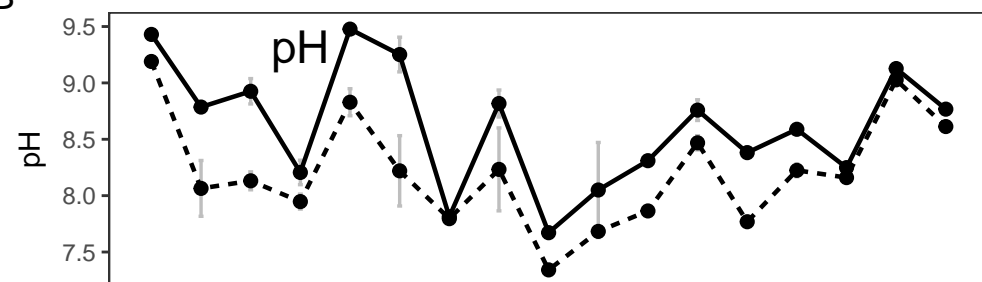

C

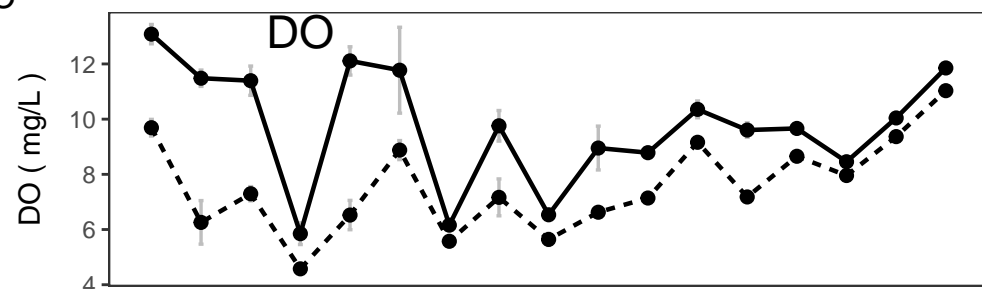

D

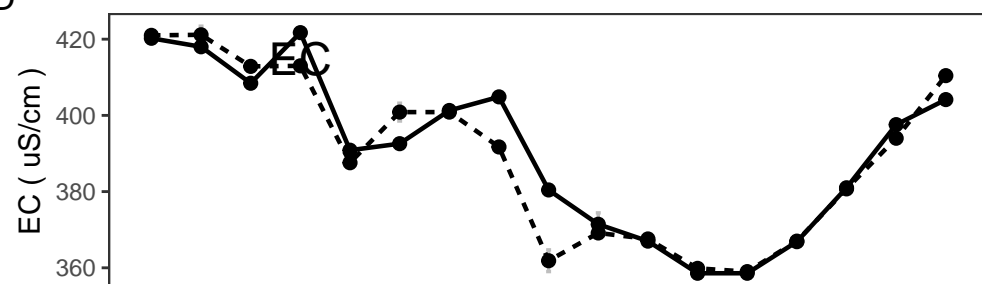

E

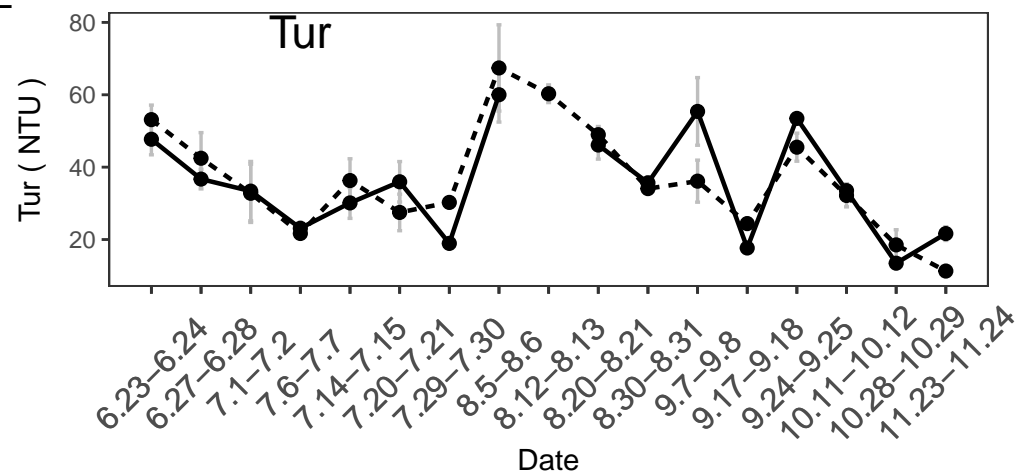

F

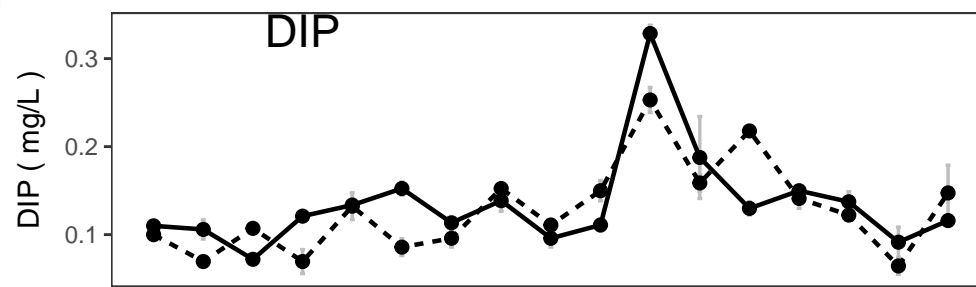

G

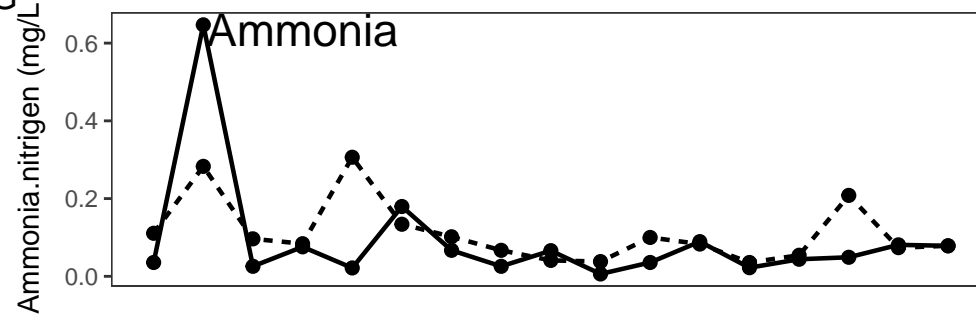

H

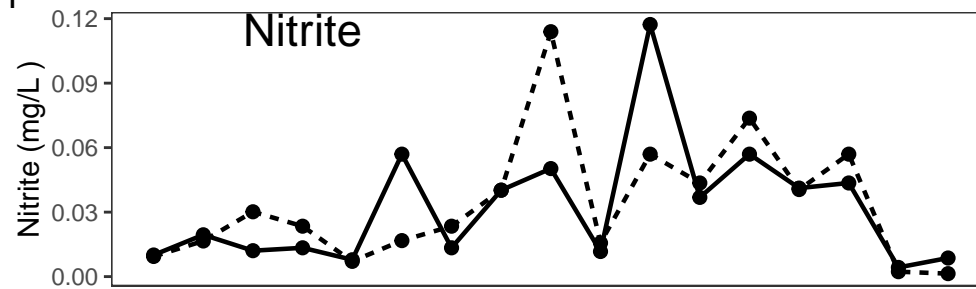

I

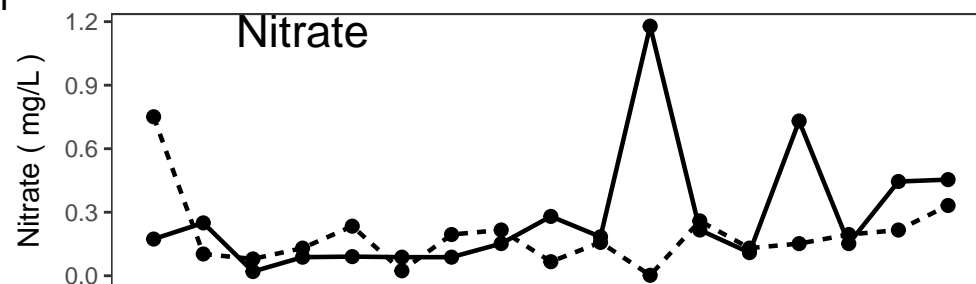

J

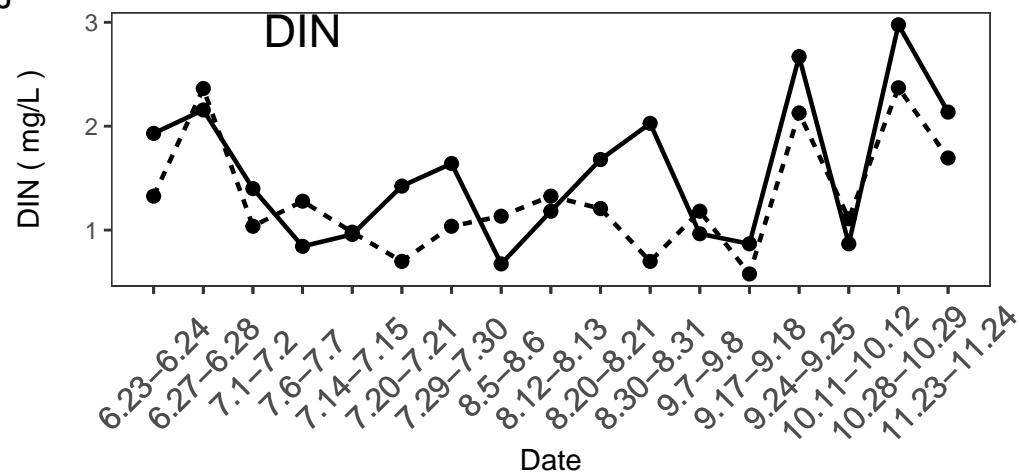

Supplement: FIG S1 [file msystems.00992-22-s0001.pdf]

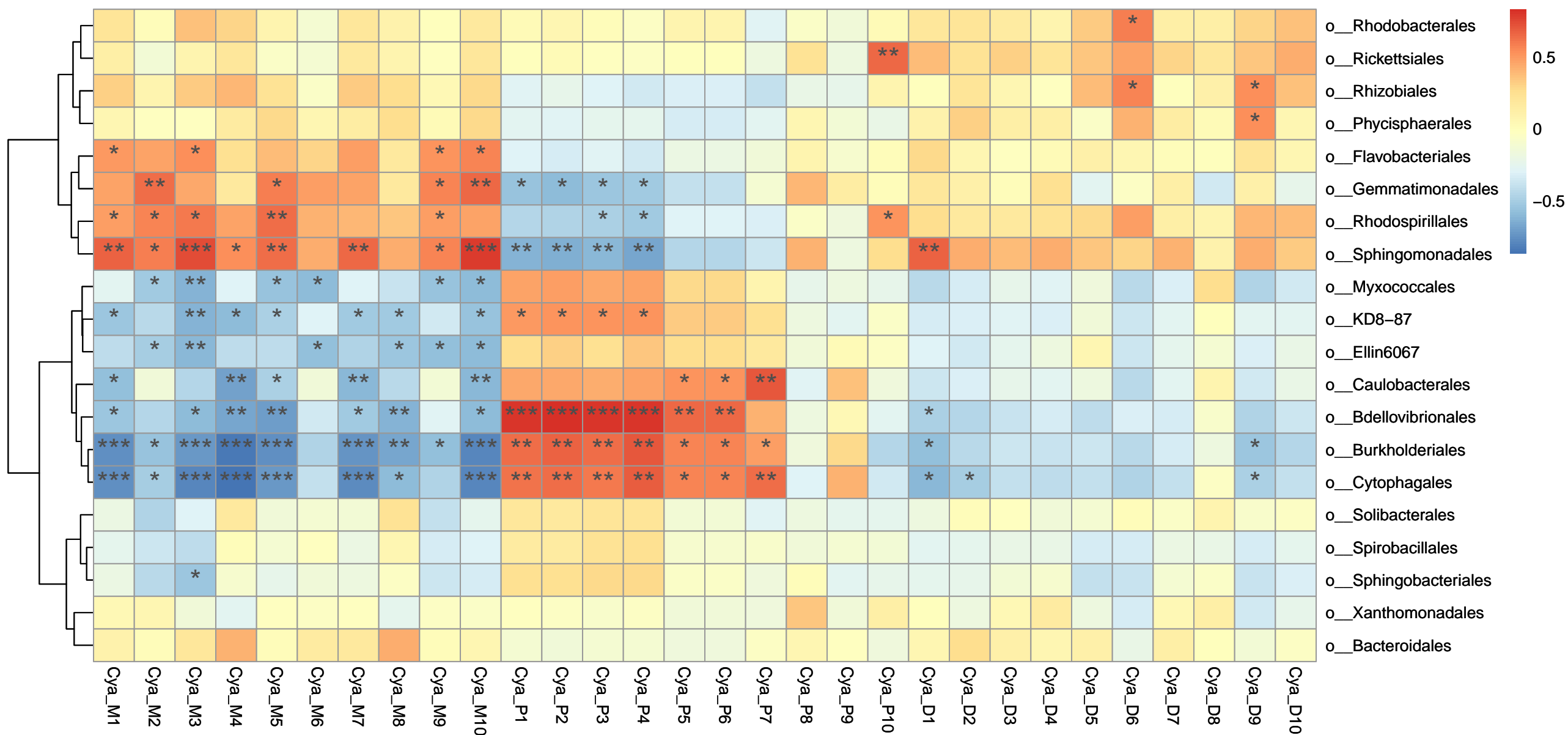

Supplement: FIG S2 [file msystems.00992-22-s0002.pdf]

A

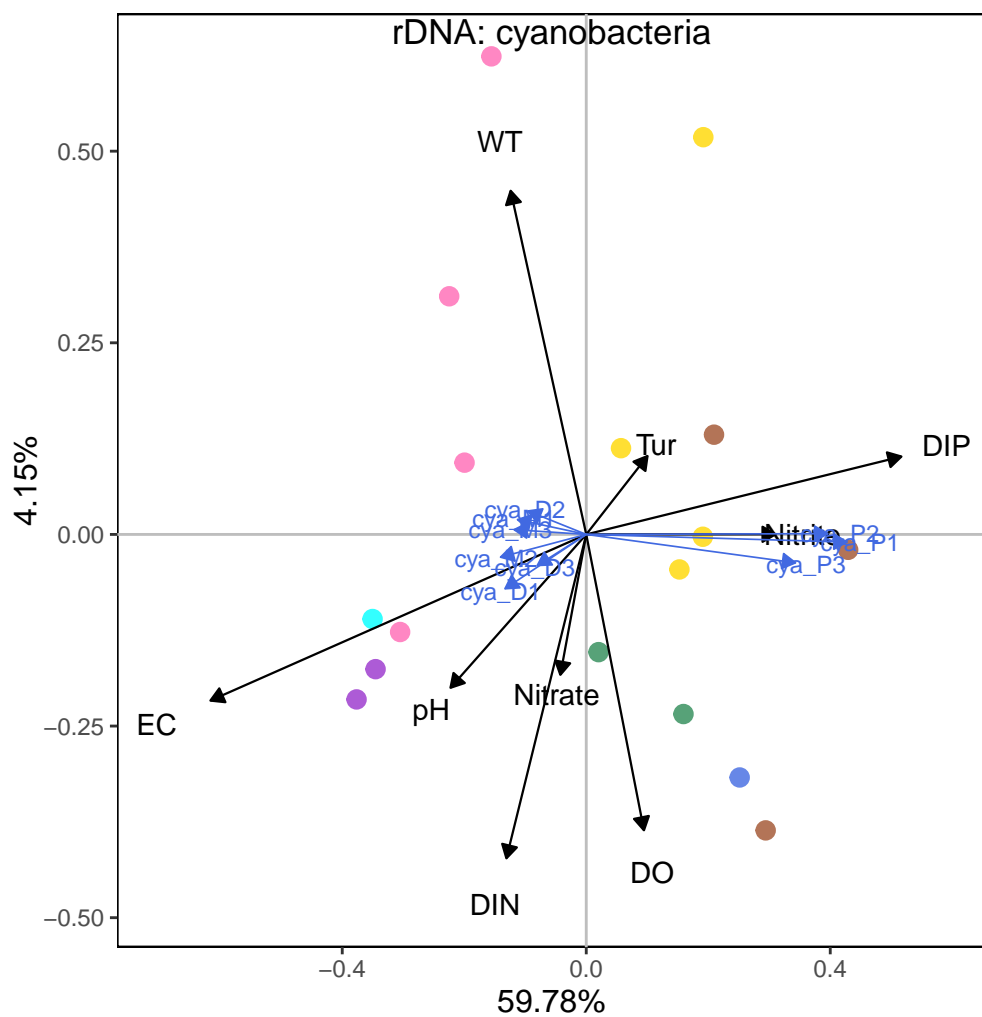

B

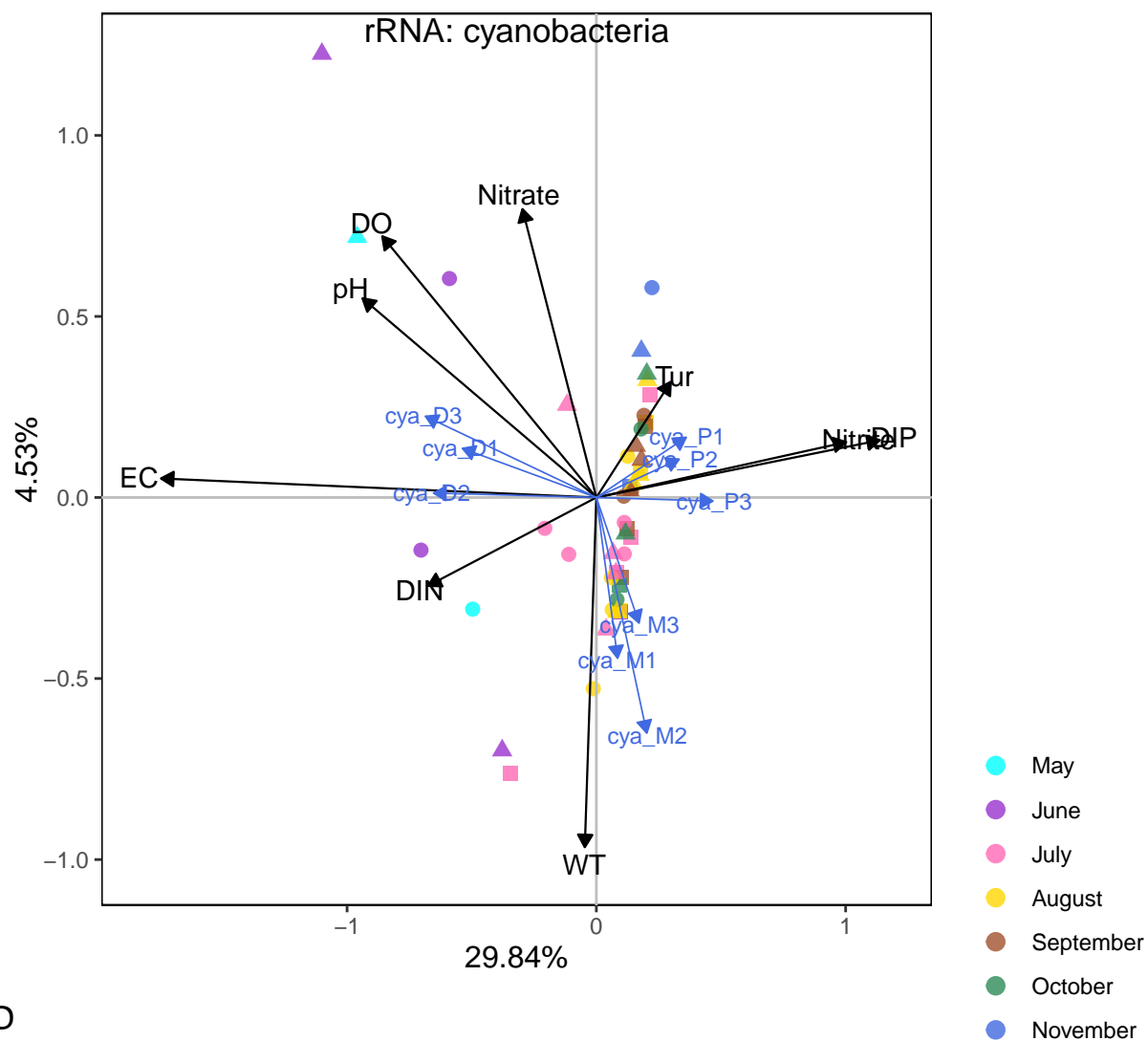

C

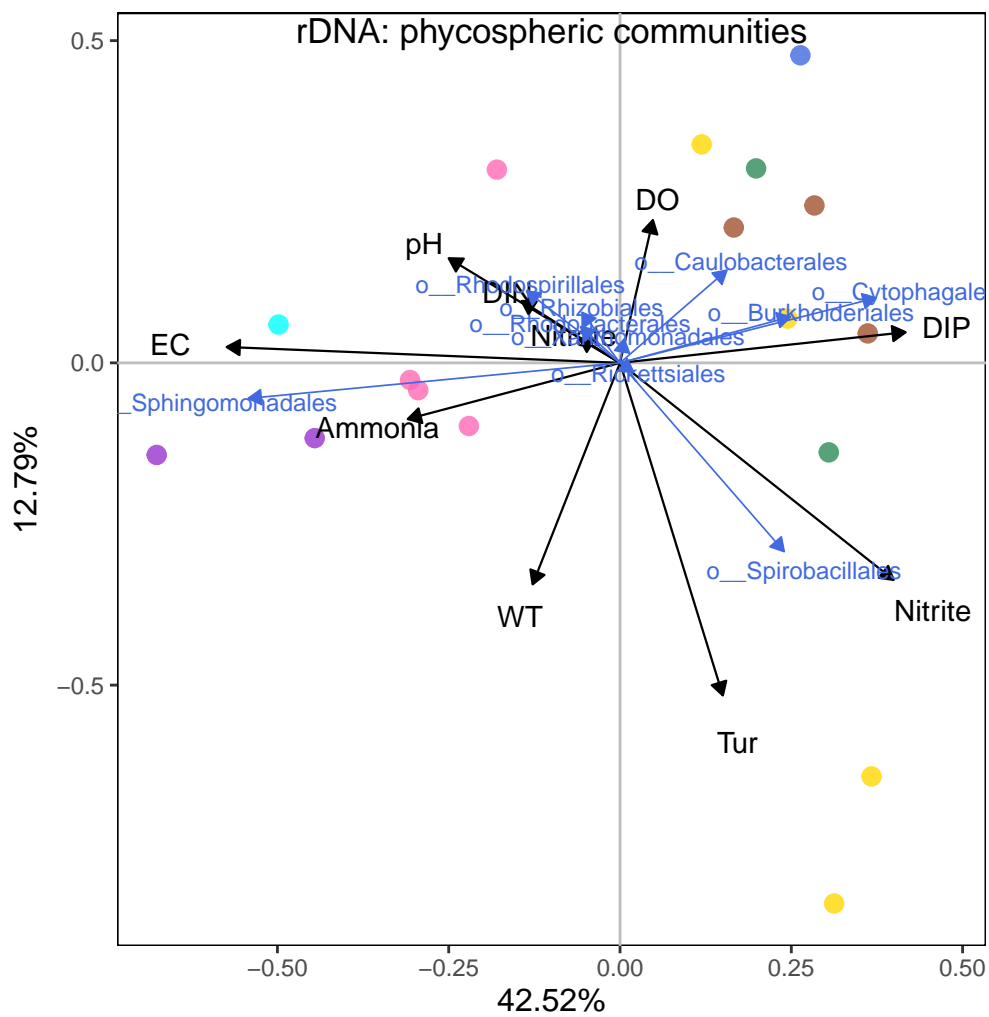

D

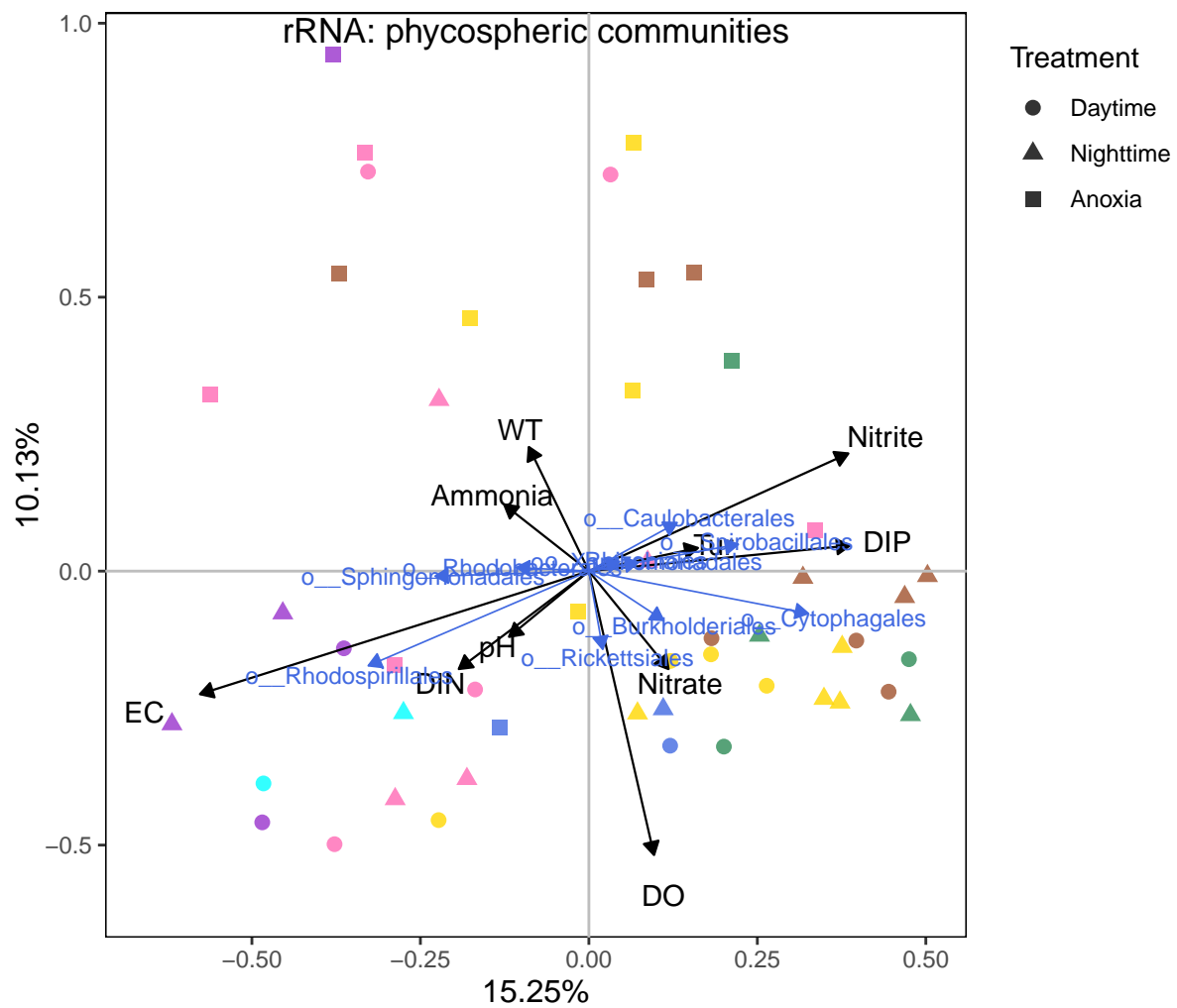

Supplement: FIG S3 [file msystems.00992-22-s0003.pdf]

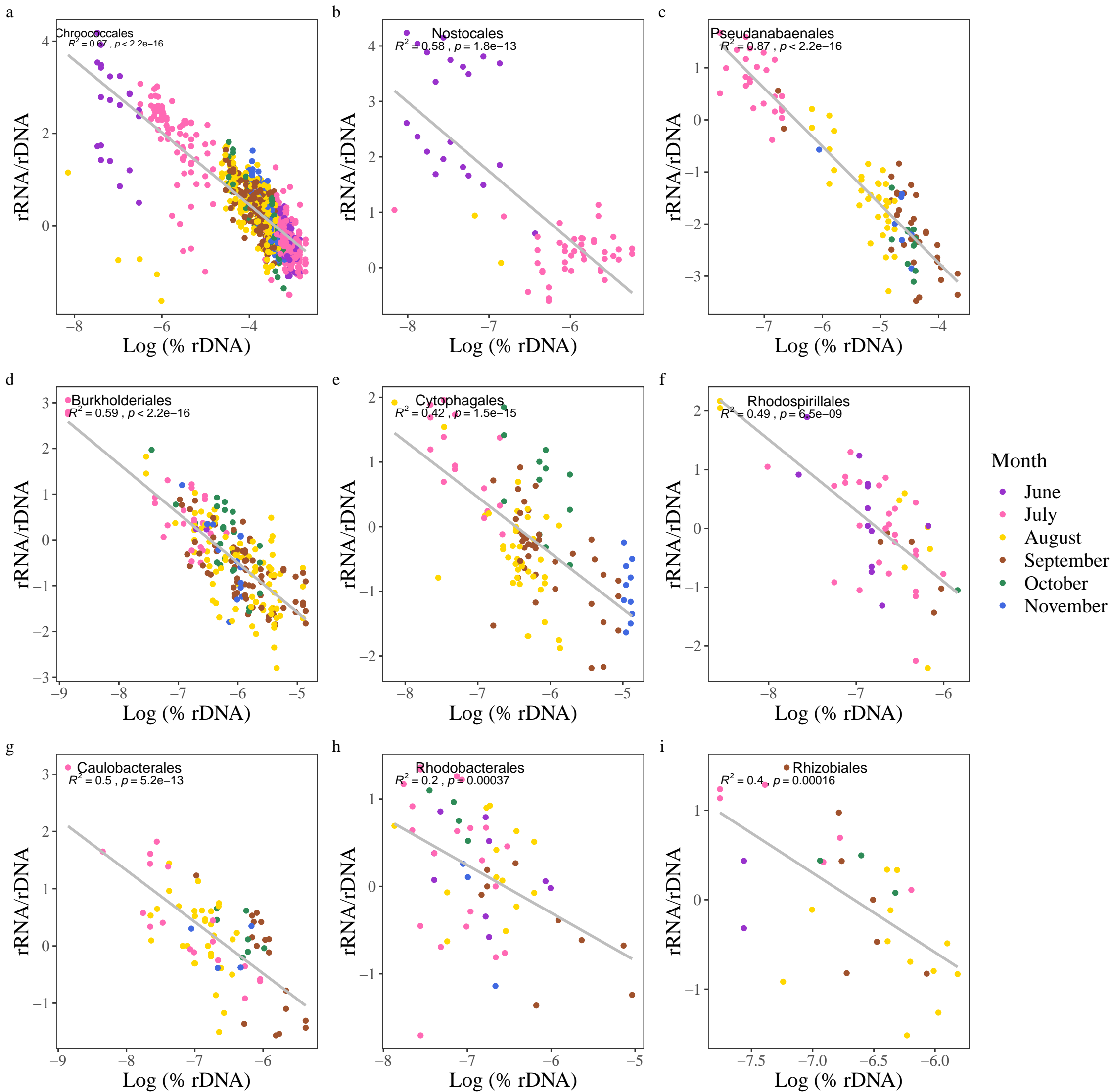

Supplement: FIG S5 [file msystems.00992-22-s0005.pdf]

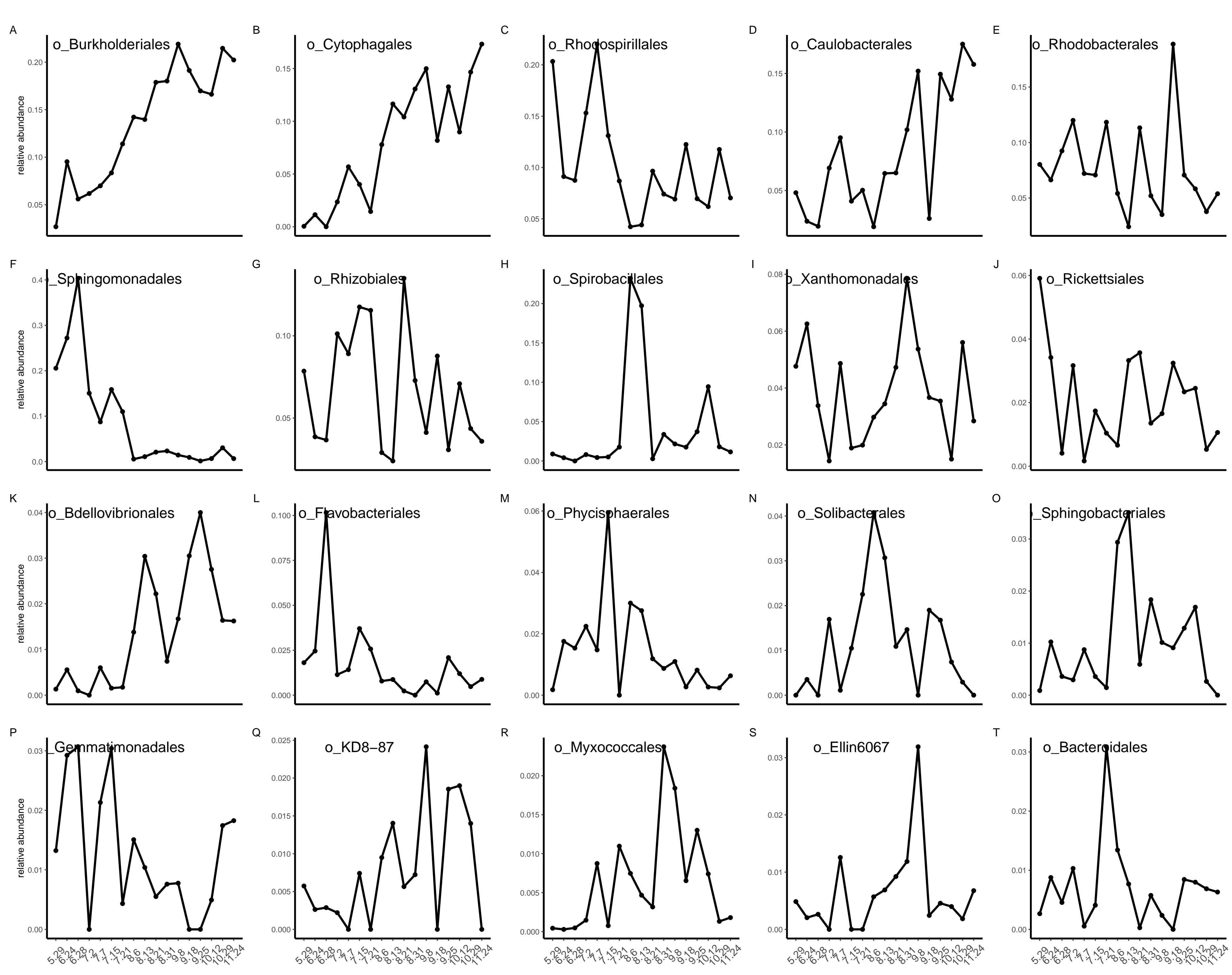

Supplement: FIG S6 [file msystems.00992-22-s0006.pdf]

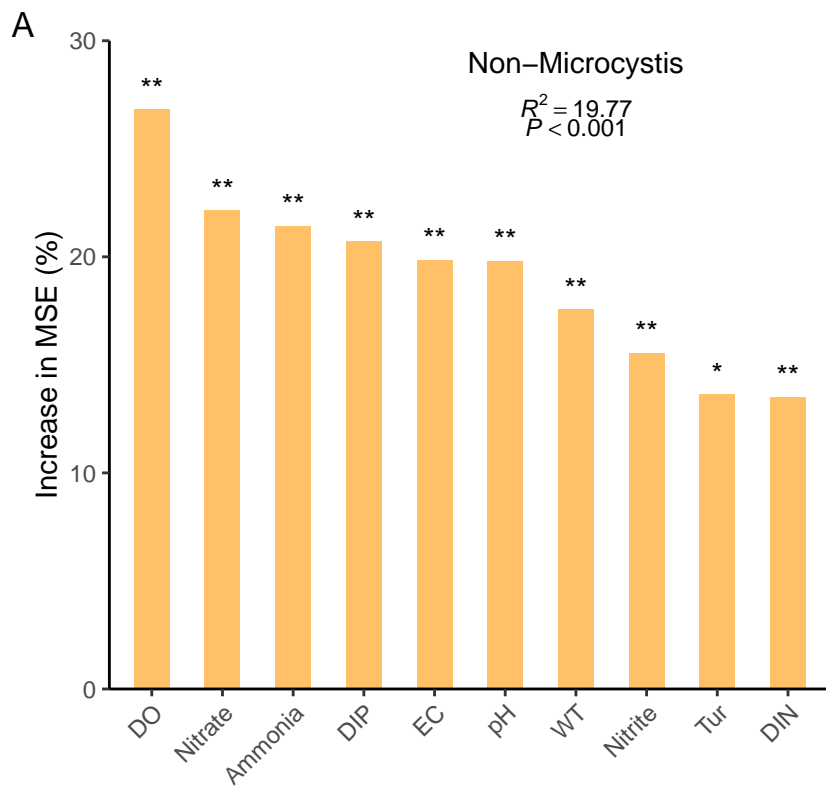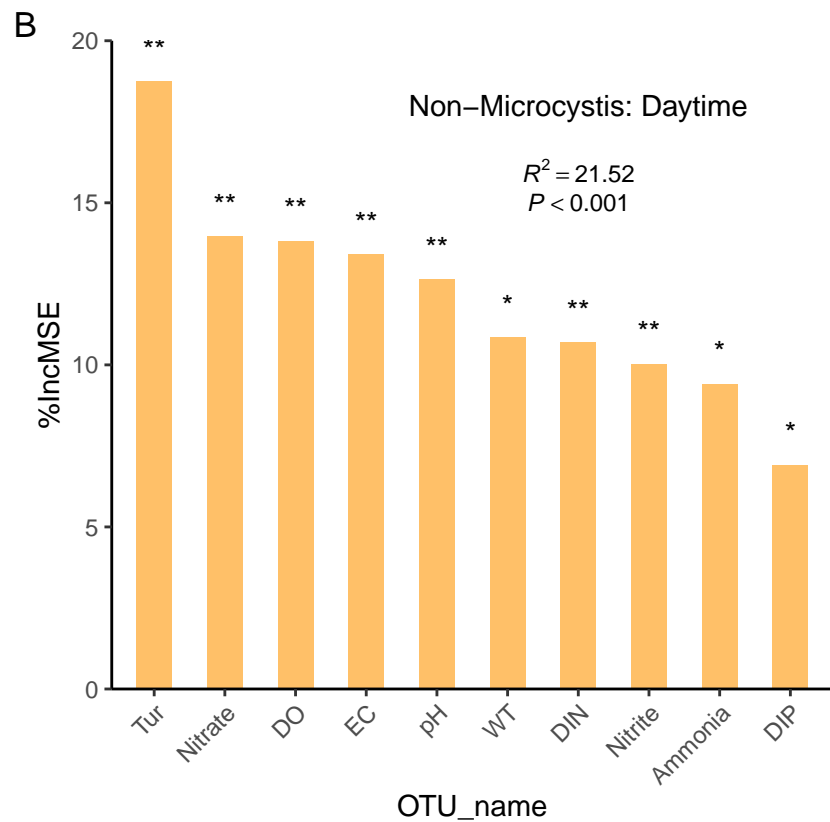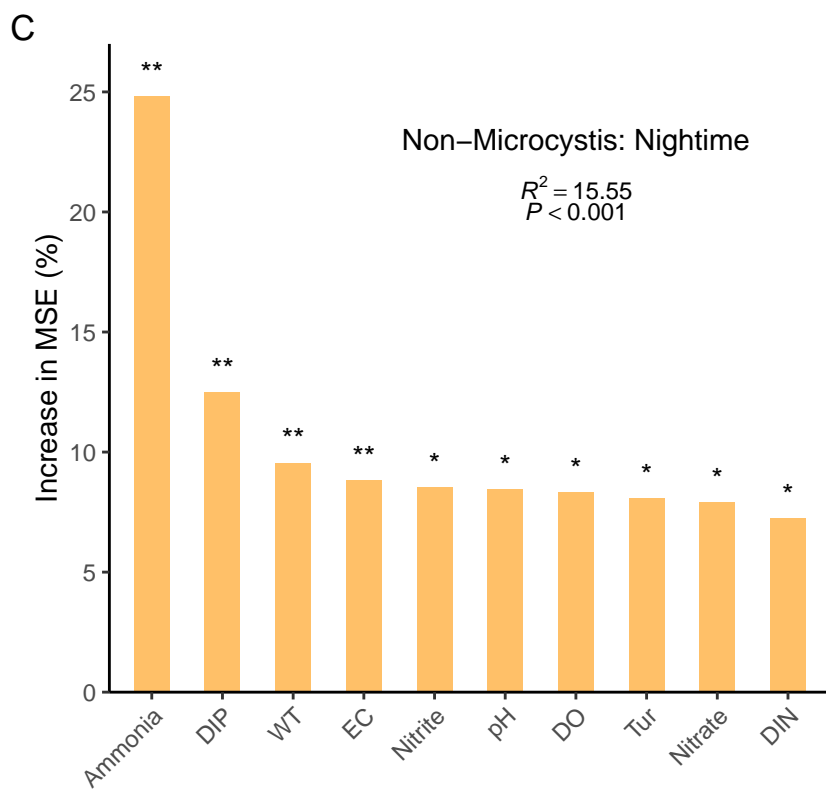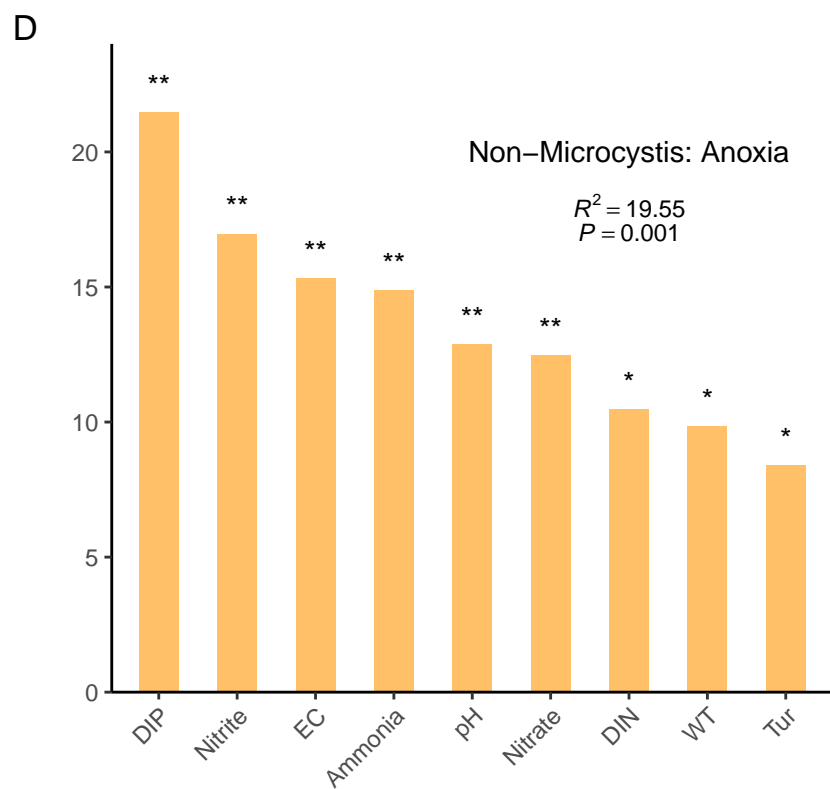

Supplement: FIG S7 [file msystems.00992-22-s0007.pdf]

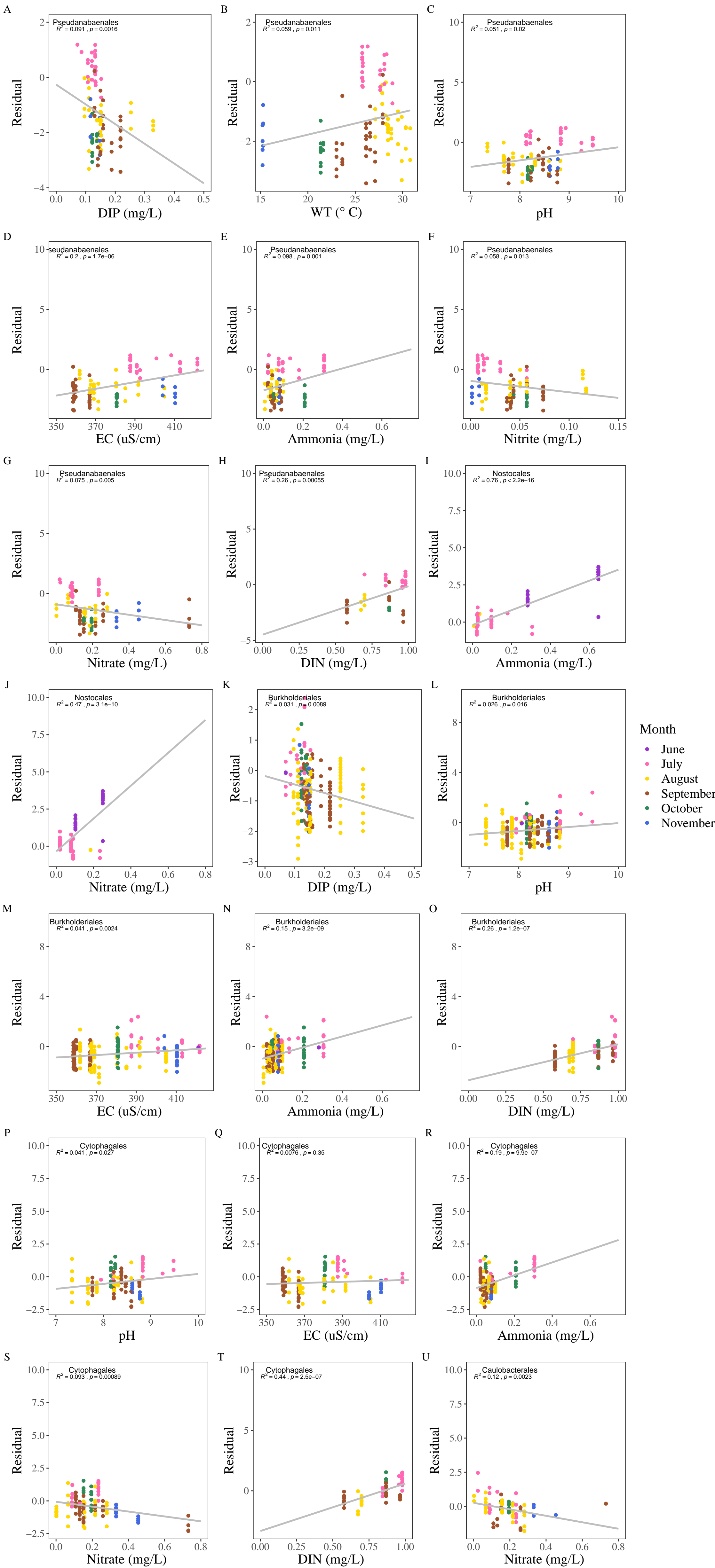

Supplement: FIG S8 [file msystems.00992-22-s0008.pdf]
