# Supplementary material for: Determinants of Total and Active Microbial Communities Associated with Cyanobacterial Aggregates in a Eutrophic Lake
Source: mSystems. 2023 Mar 16;8(2):e00992-22. doi: 10.1128/msystems.00992-22 (PMC10134853; doi:10.1128/msystems.00992-22)
Supplement: TABLE S1 [file msystems.00992-22-s0009.docx]

**Table S1.** Mantel analysis of phycospheric community composition with environmental factors and cyanobacterial community composition

|  | Mantel statistic r | *P* value |
| --- | --- | --- |
| Cyanobacteria | 0.540 | 0.001 |
| *Microcystis* | 0.517 | 0.001 |
| Environmental factors | 0.466 | 0.001 |
| Cyanobacteria (partial) | 0.492 | 0.001 |
| *Microcystis* (partial) | 0.464 | 0.001 |
| Environmental factors (partial) | 0.402 | 0.001 |
