# Supplementary material for: Determinants of Total and Active Microbial Communities Associated with Cyanobacterial Aggregates in a Eutrophic Lake
Source: mSystems. 2023 Mar 16;8(2):e00992-22. doi: 10.1128/msystems.00992-22 (PMC10134853; doi:10.1128/msystems.00992-22)
Supplement: FIG S4 [file msystems.00992-22-s0004.pdf]

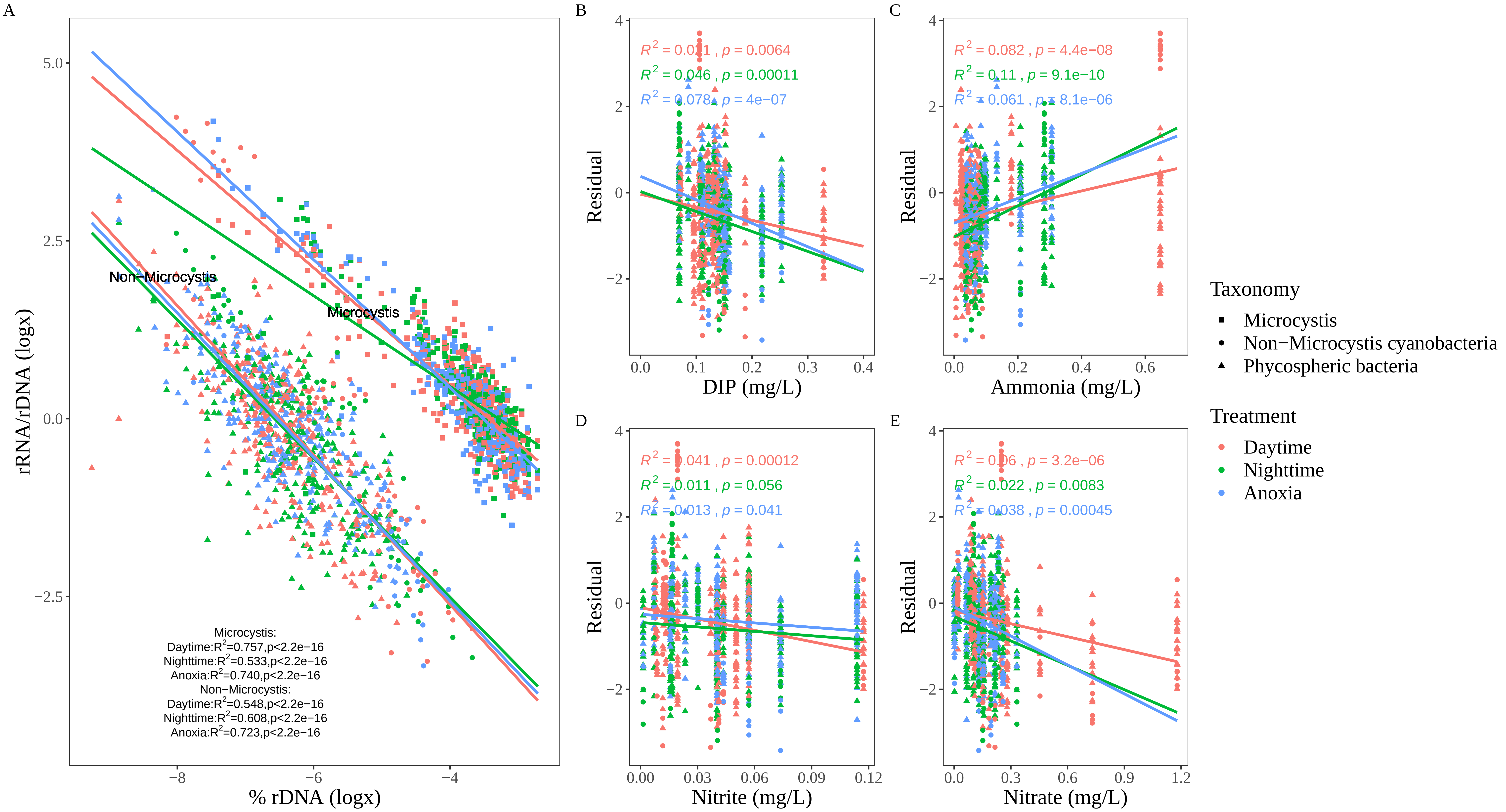

A
B
C
D
E
Non−Microcystis
Microcystis
Microcystis:
Daytime:R2=0.757,p<2.2e−16
Nighttime:R2=0.533,p<2.2e−16
Anoxia:R2=0.740,p<2.2e−16
Non−Microcystis:
Daytime:R2=0.548,p<2.2e−16
Nighttime:R2=0.608,p<2.2e−16
Anoxia:R2=0.723,p<2.2e−16
rRNA/rDNA (logx)
% rDNA (logx)
R2 = 0.041 , p = 0.0064
R2 = 0.046 , p = 0.00011
R2 = 0.078 , p = 4e−07
DIP (mg/L)
R2 = 0.082 , p = 4.4e−08
R2 = 0.11 , p = 9.1e−10
R2 = 0.061 , p = 8.1e−06
Ammonia (mg/L)
R2 = 0.041 , p = 0.00012
R2 = 0.011 , p = 0.056
R2 = 0.013 , p = 0.041
Nitrite (mg/L)
R2 = 0.06 , p = 3.2e−06
R2 = 0.022 , p = 0.0083
R2 = 0.038 , p = 0.00045
Nitrate (mg/L)
Residual
Taxonomy
Microcystis
Non−Microcystis cyanobacteria
Phycospheric bacteria
Treatment
Daytime
Nighttime
Anoxia
