# Supplementary material for: Determinants of Total and Active Microbial Communities Associated with Cyanobacterial Aggregates in a Eutrophic Lake
Source: mSystems. 2023 Mar 16;8(2):e00992-22. doi: 10.1128/msystems.00992-22 (PMC10134853; doi:10.1128/msystems.00992-22)
Supplement: TABLE S2 [file msystems.00992-22-s0010.docx]

**Table S2.** Orders with significantly different relative activities among the three conditions by MRPP analysis and paired Wilcoxon test

|  | MRPP | | | paired Wilcoxon test | | |
| --- | --- | --- | --- | --- | --- | --- |
|  | Observe delta | Expect delta | *P* value | Daytime vs Nighttime | Daytime vs Anoxia | Nighttime vs  Anoxia |
| *Chroococcales* | 0.121 | 0.121 | * | - | - | ** |
| *Nostocales* | 0.606 | 0.613 | * | ** | * | - |
| *Rhodobacterales* | 0.478 | 0.496 | ** | - | - | - |
| *Sphingomonadales* | 0.592 | 0.649 | ** | * | * | ** |
| *Flavobacteriales* | 0.609 | 0.628 | ** | - | - | - |
| *Sphingobacteriales* | 0.490 | 0.518 | ** | - | - | - |
| *Bacteroidales* | 0.613 | 0.624 | * | - | * | * |
| *Rhodospirillales* | 0.447 | 0.439 | - | - | - | *** |
| *Rhizobiales* | 0.365 | 0.373 | - | - | - | * |
